# Ultrafine and Fine Particulate Matter Inside and Outside of Mechanically Ventilated Buildings

**DOI:** 10.3390/ijerph14020128

**Published:** 2017-01-28

**Authors:** Shelly L. Miller, Nick A. Facciola, Darin Toohey, John Zhai

**Affiliations:** 1 Department of Mechanical Engineering; University of Colorado, Boulder, CO 80309, USA; Nick.Facciola@colorado.edu; 2 Department of Atmospheric and Oceanic Sciences, University of Colorado, Boulder, CO 80309, USA; Darin.Toohey@colorado.edu; 3 Department of Civil, Environmental and Architectural Engineering, University of Colorado, Boulder, CO 80309, USA; John.Zhai@colorado.edu

**Keywords:** aerosols, ultrafine particles, particle number count, infiltration, nitrate

## Abstract

The objectives of this study were to measure levels of particulate matter (PM) in mechanically ventilated buildings and to improve understanding of filtration requirements to reduce exposure. With the use of an Ultra High Sensitivity Aerosol Spectrometer and an Aerodyne Mass Spectrometer, ultrafine (0.055–0.1 μm) and fine (0.1–0.7 μm) indoor and outdoor PM was measured as a function of time in an office, a university building, and two elementary schools. Indoor particle levels were highly correlated with outdoor levels. Indoor and outdoor number concentrations in Denver were higher than those in Boulder, with the highest number concentrations occurring during summer and fall. The ratio of indoor-to-outdoor (I/O) PM was weakly but positively correlated with the amount of ventilation provided to the indoor environment, did not vary much with particle size (ranged between 0.48 and 0.63 for the entire size range), and was similar for each period of the week (weekend vs. weekday, night vs. day). Regression analyses showed that ultrafine indoor PM baseline concentrations were higher at night from nighttime infiltration. A lag time was observed between outdoor and indoor measurements. Weekday days had the shortest lag time of 11 min, and weekend nighttime lags when the HVAC was not in use were 50 to 148 min. Indoor-outdoor PM concentration plots showed ultrafine PM was more correlated compared to fine, and especially when the HVAC system was on. Finally, AMS data showed that most of the PM was organic, with occasional nitrate events occurring outdoors. During nitrate events, there were less indoor particles detected, indicating a loss of particulate phase nitrate. The results from this study show that improved filtration is warranted in mechanically ventilated buildings, particularly for ultrafine particles, and that nighttime infiltration is significant depending on the building design.

## 1. Introduction

Because people spend upwards of 85% of their time indoors, it is widely recognized that a significant portion of total personal exposure to particulate matter (PM) occurs in indoor environments. Roughly 80% of the time indoors is spent in residences, while the other 20% is spent in offices, restaurants, schools, and other indoor locations that are mostly mechanically ventilated buildings. Indoor particle concentrations are often lower in mechanically ventilated buildings compared to outdoor particle concentrations, since most buildings have some filtration in their ventilation systems, and there are limited indoor sources [1,2,3,4]. This research was conducted to better understand the factors that influence PM in mechanically ventilated buildings.

Outdoor mass concentrations of PM_2.5_ (particulate matter with diameters < 2.5 µm) have been strongly associated with an increased risk of acute lower respiratory infections [5] and mortality [6,7,8,9]. Adverse respiratory health effects and an increase in mortality has also been associated with ultrafine PM (particulate matter with diameters < 0.1 µm) [10,11,12,13]. Studies of ultrafine and fine PM in mechanically ventilated buildings show that the indoor mass concentrations are less compared to outdoor concentrations, except when strong indoor sources are present [4,14]. Particulate matter in buildings comes from outdoors, infiltrating through the building envelope or entering directly through the ventilation system, from resuspension of indoor particulate matter due to occupant movement, or from indoor sources. Outdoor particles can enter buildings through the building envelope if the building is not airtight and there is a driving force such as temperature or pressure differences. Data from an analysis of 139 buildings around the world (90 in the US) showed that these commercial buildings were not airtight and some were actually quite leaky [15]. Liu and Nazaroff [16] report the fraction of fine particles with diameters in the range 0.1–1.0 μm that penetrate through the building envelope is nearly unity.

Most commercial buildings draw outside air in through the ventilation system, conditioning and filtering the air and mixing it with return air. The general practice in commercial buildings is to install low-efficiency filters with a Minimum Efficiency Reporting Value (MERV) rating from 4 to 8 [14,17,18]. These filters do not efficiently remove particles less than 3 μm. The higher the MERV rating the more efficient the filter is at removing particles, particularly smaller particle sizes.

Occupant activities or indoor sources can elevate particle levels indoors. Studies have shown minimal importance of particle resuspension on indoor submicron PM concentrations in residences [19,20]. Wu et al. [14] measured higher indoor-outdoor concentration ratios for particles greater than 0.5 μm in daycares, likely due to children playing. Most commercial buildings do not have indoor sources of particles. Exceptions include buildings that house restaurants, hair salons, and dental offices [14]. The objective of this study was to expand upon previous studies of particles in commercial buildings by measuring the PM concentration as a function of size and chemical speciation. These data inform understanding of commercial building filtration requirements and potential indoor exposures of building occupants. Measurements were made over a year to elucidate any seasonal differences. Buildings were studied in two locations, the large urban city of Denver and the smaller, more rural town of Boulder, Colorado. Instruments were set up to collect data inside and outside of two schools, one university building, and one office building.

## 2. Materials and Methods

### 2.1. Testing Sites

Denver and Boulder, two cities in Colorado (USA), were chosen for this study. Denver (population 650,000) is a heavy metropolitan area with major high motor traffic areas including major interstate freeways and industrial facilities. Boulder (population 100,000) is a light metropolitan area with very few heavy industries, one major interstate highway and one Colorado highway. Boulder is 30 miles northwest of Denver on the easternmost foothills of the Rocky Mountains. Four buildings were enrolled in this study, two in Denver and two in Boulder. The buildings where chosen because of their location, willingness to participate, and the heating, ventilating, and air-conditioning (HVAC) system design and accessibility.

The Denver School of the Arts (DSA), built in 2004, is in a neighborhood located near (1 km) a major roadway. The air sampling was done on the second floor. Two sampling tubes were routed from the instrumentation in the mechanical room into the false ceiling. One tube was dropped into the science room and the other went beyond and into the hallway. The third line was routed through the air intake vent to the outside of the building. The outside line rested 0.6 m away from the edge of the exterior wall, approximately 6 m above the ground.

The Denver three-story multipurpose building office building is located on Grant Street, built in 1960. The office building is approximately 8 km west of Denver School of the Arts, in the downtown area but not far from I-25 and I-70. Indoor sampling at this building was conducted in offices on the third floor. The outside sampling line was slipped through a north exterior window, which was afterwards sealed with duct tape to avoid infiltration. The sampling equipment was located in an office room separate from those being sampled.

In Boulder, the Eisenhower Elementary School, built in 1971, is in a neighborhood located 300 m to the nearest major road and 1.6 km east of the I-36. The two indoor sampling locations selected for this building were the music classroom and the teacher’s lounge. The sample tubes were hung from the false ceiling, near the entry doors. The sampling location was stored in the janitor’s storage room, across the hallway from the music room. The outside line was routed through the adjacent boiler room and out the vents to the outside.

The Integrated Teaching and Learning Laboratory (ITLL) built in 1997, is located on the University of Colorado campus adjoining the Engineering Center. This is 2.4 km west of Eisenhower Elementary, 122 m from I-36. For this study, one indoor line was hung above the main level laboratory plaza; the other indoor line was routed to the stairwell between the first and second floors of the building. The outdoor line was routed to the HVAC intake plenum on the roof, sampling air just before it entered the air-handling unit. All sampling equipment was kept in the mechanical room.

A summary of the HVAC system and the filters’ Minimum Efficiency Reporting Value (MERV) used in each building is given below in Table 1. Additional details of the buildings and HVAC systems are available [21].

### 2.2. Experimental Setup

**Figure 1:**
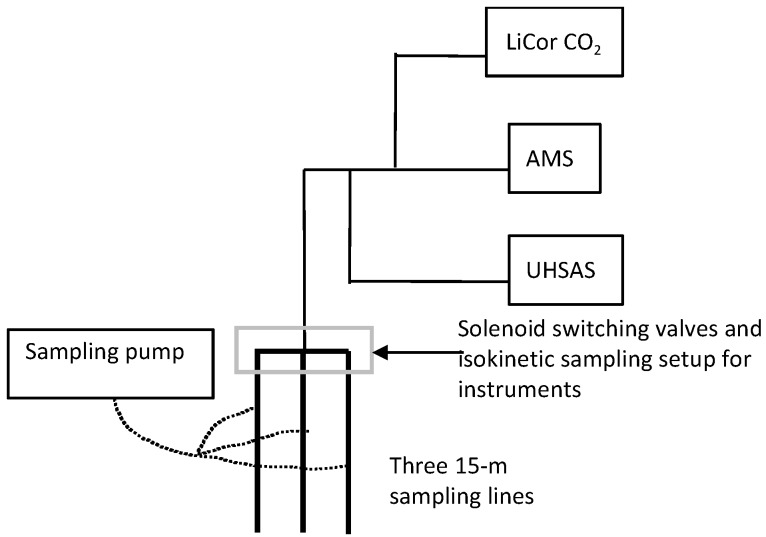
Experimental setup for sampling in each study building.

Figure 1 details the experimental setup. For each of the buildings, three sample lines were routed such that two indoor locations and one outdoor location could be reached from an appropriate equipment storage location. Each line was a 15-m, 1.3-cm nominal diameter copper tube. The sampling inlet was split with a solenoid-switching valve that switched between the three lines every four minutes. Air was drawn through the 15-m lines with a pump, drawing approximately 12 L·min^−1^ through each tube. This ensured updated concentration readings directly following a line-switch. Before the air was drawn through the pump, a smaller inlet with a much smaller flow (approximately 1.0 L·min^−1^) sampled the air. This inlet was positioned where the switching valves were located. Finally, after the three sampling lines merged to a single one, it was split again to the three sampling instruments via ¼-inch copper tubing. Where the flow changed before the pump, the flow rates were adjusted to ensure isokinetic sampling, although submicron particles are hardly affected by anisokinetic sampling. The variables available to adjust these flow rates were the wide range of possible flow through the CO_2_ analyzer as well as a flow restrictor before the large pump. The copper tubes were grounded to avoid electrostatic forces from charged particles.

The Ultra High Sensitivity Aerosol Spectrometer (UHSAS, Particle Metrics, Inc., Boulder, CO, USA) is an optical-scattering laser-based aerosol particle spectrometer for accurately and precisely sizing particles in the range of 55 nm to 1.0 μm in diameter. A major advantage of the UHSAS is its high resolution. The time resolution can be set to as fast as one second and the diameter resolution as low as 1.6 nm. The UHSAS was set to count and save the number histogram every ten seconds to avoid its upper count limit. The 100 size bins were set on a logarithmic scale over its full range. The flow rate was set to 24 cm^3^/min. Measurements from this instrument for diameters 55 nm to 0.1 µm are referred to as ultrafine and for diameters 0.1 to 0.7 µm as fine PM. Data above 0.7 µm was deemed too noisy due to low number counts.

The Aerodyne aerosol mass spectrometer (AMS) is a fast and reliable instrument for continuous monitoring of fine aerosol non-refractory chemical composition and size-resolved particle mass distributions as a function of particle composition. The AMS has been described in detail elsewhere [23,24]. For the particular AMS used in this experiment, the sample flow rate is restricted by a critical orifice inlet with a diameter of 100 μm, providing a flow of 85 cm^3^·min^−1^. This instrument was set to save every four minutes, alternating 5 s on the mass spectrum mode and 25 s on the time-of-flight mode.

The AMS was size calibrated prior to usage at each test site and the ionization efficiency was calibrated a few times throughout that week. To generate ammonium nitrate particles for AMS size calibration, a pump first pushed air through a filter, then into a Model 3076 Constant Output Atomizer (TSI, Inc., Shoreview, MN, USA) in combination with a TSI, Inc. Model 3062 Diffusion Dryer. The polydisperse aerosols were sized to a mobility diameter of 350 nm using a TSI Inc. Model 3080 Electrostatic Classifier with a Model 3081 Long Differential Mobility Analyzer (DMA). The generated aerosol was transported via ¼ inch copper tubing directly into the AMS inlet. The AMS was deployed at only the DSA and ITLL due to storage requirements.

The concentration of CO_2_ in the indoor and outdoor locations was measured using a LI-7000 CO_2_/H_2_O Gas Analyzer (Li-Cor, Lincoln, NE, USA). It offers a 1% accuracy of CO_2_. The time resolution of the CO_2_ analyzer for this study was set to 0.2 Hz and the flow was 1000 cm^3^·min^−1^, as it was the free factor for ensuring isokinetic sampling at the point where the air flow rate changes in the sample tubes.

Average line losses in the sampling system and instrumentation setup were estimated with the UHSAS. The average losses across the entire size range were 20%–30%. The total loss from the entire sample line configuration was 22.4% ± 7.1% by number and 23.5% ± 8.7% by mass. Since the focus of this study was on the difference between indoor and outdoor PM concentration values rather than the absolute magnitudes, this line-loss error was not accounted for in the data analysis; the losses influenced the values from all three lines equally and were on average uniform across particle sizes.

Testing dates are summarized in Table 2. Data was collected for 4–8 days, ensuring the collection of two 24-h periods during a weekday and a weekend at each test site. Two times during the summer, the UHSAS and AMS did not collect representative data, because of flow and leak problems. These instances called for a second week of sampling to obtain the missing data. Without enough summer time left to sample again, the data during the summer at the Eisenhower Elementary School in Boulder was deemed unusable.

### 2.3. Air Exchange Rates

Before each sampling campaign at each building, the air exchange rates (AER) were assessed for the sampling room using a tracer gas test protocol with CO_2_. The CO_2_ tracer gas method provides only the AER for the room at the time it was conducted. This was done by releasing CO_2_ into the room from a high-pressure CO_2_ tank, until a maximum concentration of 5000 ppm was reached in the room. Once the desired concentration was reached, the CO_2_ tank was closed and the natural decay was recorded until background levels (~450 ppm) were reached. To find the AER, the natural logarithm of the CO_2_ was plotted against the time in hours. Only data > 1000 ppm CO_2_ were used in this analysis. The slope of the linear fit through this data is the AER in air changes per hour (1/h).

The AER was also calculated by dividing the interior volume of the space by the volumetric supply airflow rate. The supply airflow into the sample rooms was measured using an ALNOR (Shoreview, MN, USA) Standard Bolometer Capture Hood, by measuring the volumetric airflow rate from the supply diffusers.

Three methods were used to find the percentage of fresh air in the supply air. At the Denver school, a direct readout of the fresh air damper position was logged by the HVAC department of Denver Public Schools computer system. The University building had an online sensor system that allowed for acquiring data about the HVAC system, including the flow rates of the supply, fresh, return and exhaust air streams. The percentage of fresh air in the supply air was found by dividing the fresh airflow rate by that of the supply air.

The remaining two buildings do not have this kind of online computer technology, and thus a different method was required. Temperature sensors were placed in the HVAC ducting to record the temperature of the outside air, return air, and mixed air. The sensors used were portable, battery-powered temperature data loggers. Equation (1) was used to find the fresh air make-up, based on the principle that the mixed air ($T_{mixed}$) will have a temperature that is either closer to the outside air ($T_{outside})\;$or return air ($T_{return}$), depending on which is being used for supply air in greater quantity. Since the two rooms at Eisenhower Elementary School are on different zones, two sets of temperature loggers had to be used in the separate air-handling units:

(1) $$Fresh\;Air\;\%=\;\frac{T_{mixed\;}-\;T_{return}}{T_{outside}-\;T_{return}}\;\times \;100\%$$

This method can be unreliable because the temperatures are influenced by external factors such as the sun directly radiating on the outside sensors, or the time it takes for the temperatures to adjust when the damper position changes. However, with careful examination of the temperature data, the general opening and closing of the fresh air damper can be assumed.

## 3. Results and Discussion

### 3.1. Air Exchange Rates

The results for the space AER using the tracer gas protocol method and the supply airflow rate method are given in Table 3. The AER for the teacher’s lounge at the Boulder school was exceedingly high, possibly due to a design airflow rate that accounted for cigarette smoking, as the building was built in 1971. Each air exchange rate test was conducted during the day when the HVAC was running, except for the single overnight run at the Boulder school. This was done in the music room with the purpose of establishing a typical nighttime air exchange rate.

### 3.2. Concentrations and Indoor/Outdoor Ratio as Function of Particle Size

**Figure 2:**
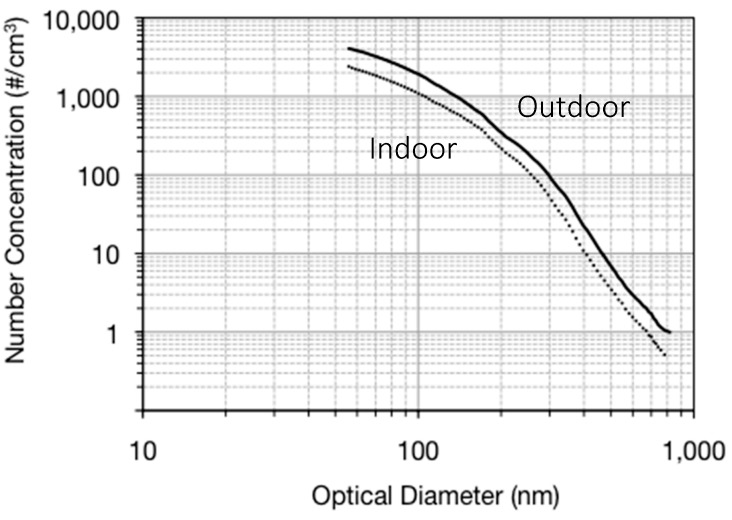
Indoor and outdoor averaged number concentrations for the entire sampling year, as a function of optical diameter (four buildings, four seasons, and 4–8 sampling days per season).

**Figure 3:**
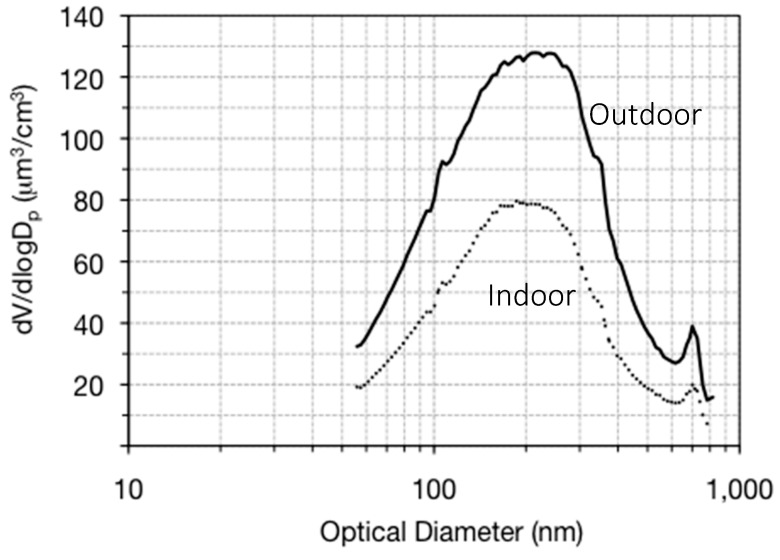
Indoor and outdoor averaged volume concentration size distribution for the entire sampling year, as a function of optical diameter.

Over all sixteen datasets, the outdoor PM concentrations were generally higher than the indoor concentrations, with the exception of a few indoor source occurrences. Figure 2 shows the overall indoor and outdoor number concentration averages from the entire dataset. The total outdoor number concentration averaged across size bins for the entire data set was 88,690 particles/cm^3^. The total indoor number concentration averaged across size bins for the entire data set 51,370 particles/cm^3^. Figure 3 shows the yearly averaged volume concentration size distributions. The peak volume occurred at 230 nm for the outdoor average and 200 nm for indoors (volume is being used as a proxy for mass).

The overall averaged outside PM number concentrations in Denver were slightly higher than those in Boulder. The indoor particle concentrations were also higher in Denver compared to Boulder, and especially for diameters less than 150 nm. Plots of the indoor and outdoor number concentration as a function of particle diameter (Section A Figures S1–S15), and the total number concentration for diameters < 0.7 µm (Section B Figures S16–S30), measured with the UHSAS for all sampling periods in all buildings can be found in the Supplemental Data.

**Figure 4:**
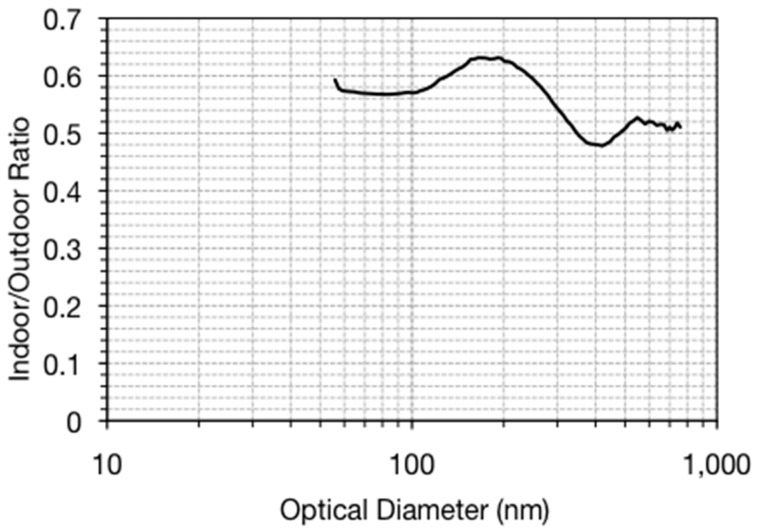
Indoor/outdoor ratio for the entire sampling year, as a function of optical diameter.

Figure 4 shows the yearly-averaged indoor/outdoor ratio (I/O) as a function of particle diameter. The ratio range is small, from 0.48 to 0.63 across the entire size range and the mean ratio across the entire size range is 0.56. The average I/O had a local maximum at a diameter of 165 nm and a minimum at 397 nm. Some outdoor particles are being removed, or “filtered out”, as they move indoors; thus, the increase at 165 nm can be explained by typical filter efficiencies dropping near this size, the size that is too large for diffusion to be effective and too small for impaction or interception to be effective [25,26]. The average I/O was similar for Denver and Boulder: 0.56 and 0.53 respectively. Section C of the Supplemental Data details all of the I/O data (Tables S1, S3, S5 and S7).

**Figure 5:**
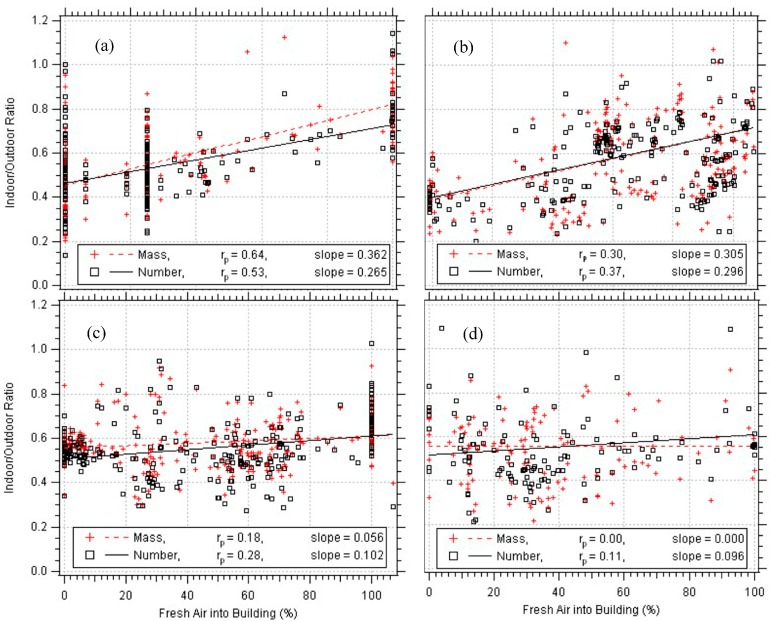
Indoor/outdoor ratios by mass and number, as a function of fresh air intake for the (**a**) Denver school; (**b**) university; (**c**) office building; and (**d**) Boulder school. Pearson’s correlations (r_p_) and slopes from linear regression analysis are presented.

The fresh air intake and the I/O were positively correlated (Figure 5). The Denver school (Figure 5a) and the university building (Figure 5b) had the highest correlations and slopes showing a stronger dependency of fresh air intake on the ratio. In a mechanically ventilated building, one would expect that when the HVAC unit brought in more air from the outside, the I/O would increase and approach a value of unity. Conversely, the I/O would drop when the HVAC unit was recirculating the inside air or was turned off. These results suggest that for the buildings with lower correlations and slopes outdoor particles are either penetrating the building through other unintentional openings, and not necessarily being brought in by the HVAC system, or there are indoor sources.

The only other parameter that showed some influence on the I/O was the wind speed. It was found that the mass ratio (r_p_ = 0.19) correlated better than the number ratio (r_p_ = 0.12). Because the number concentration is dominated by the ultrafine particles and the mass concentration by the larger fine particles, this result suggests that the wind has minimal effect on outdoor submicron particle resuspension. The school in Denver had the highest relation between the two (r_p_ = 0.32), followed by the school in Boulder. The university and office building had low correlation between the I/O and wind speed. The summer showed the highest correlation (r_p_ = 0.36), followed by fall and winter. Other parameters that were investigated for I/O correlation were temperature, relative humidity, and wind direction. None of these showed any influence on I/O.

### 3.3. Seasonal, Weekly, and Daily Trends

**Figure 6:**
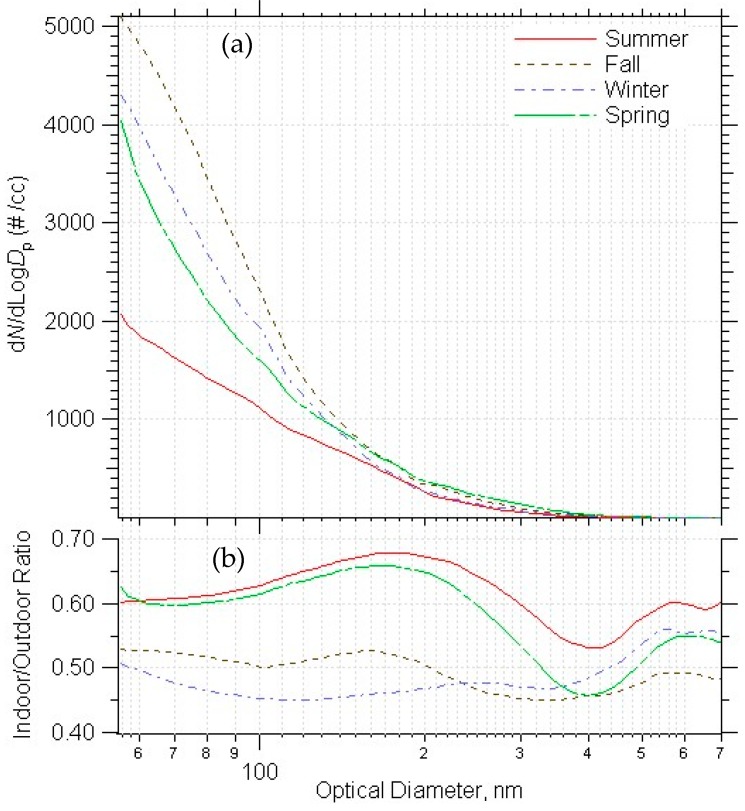
Ambient PM number distribution (a) and indoor/outdoor ratio (b) averaged by season.

The fall had the highest outdoor number concentrations, followed by winter, spring then summer (Figure 6a). Higher PM concentrations during winter has been consistently observed [27,28,29]. The ultrafine indoor/outdoor number ratio (for particles < 300 nm) was particularly affected by season; it was highest during the summer and spring (Figure 6b). This could be accounted for by the opening of windows (for those buildings with opening windows), the propping-open of doors, or more fresh air being brought inside with the HVAC system during these seasons.

The data was analyzed as a function of day/night and weekday/weekend. The reasons these time periods might exhibit different particle levels are building occupancy, differences between weekday/weekend traffic, and HVAC usage, among others. With these four mechanically ventilated buildings, a more appropriate way to break down the data is by times when the HVAC system was usually turned on (weekday daytime), usually turned off (weekday and weekend nighttime) and when it was sometimes turned on (weekend daytime). The daytime period was 6 am–8 pm, when the HVAC system was usually running at all four buildings.

**Figure 7:**
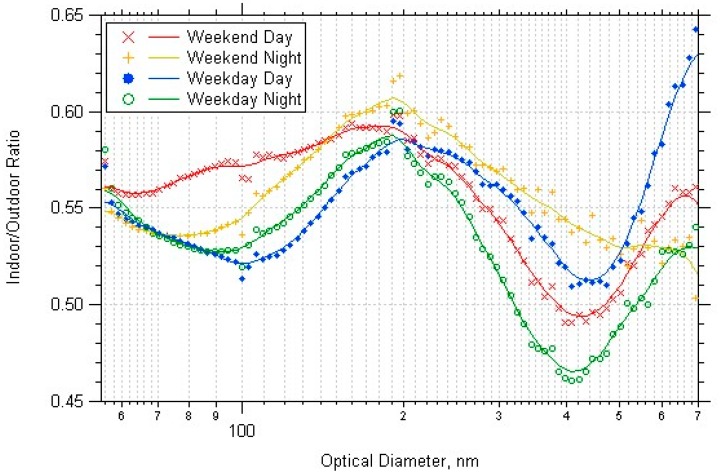
Indoor/outdoor ratio averaged over all datasets, split by period of week.

Figure 7 shows the I/O for each period of the week, averaged over all four seasons. For diameters 400–700 nm the ratio for weekend-nighttime did not increase as particle size increased, which might be because there were no indoor sources during this time period. The other time periods would have had more indoor sources of particles, such as people in the building working or cleaning.

**Figure 8:**
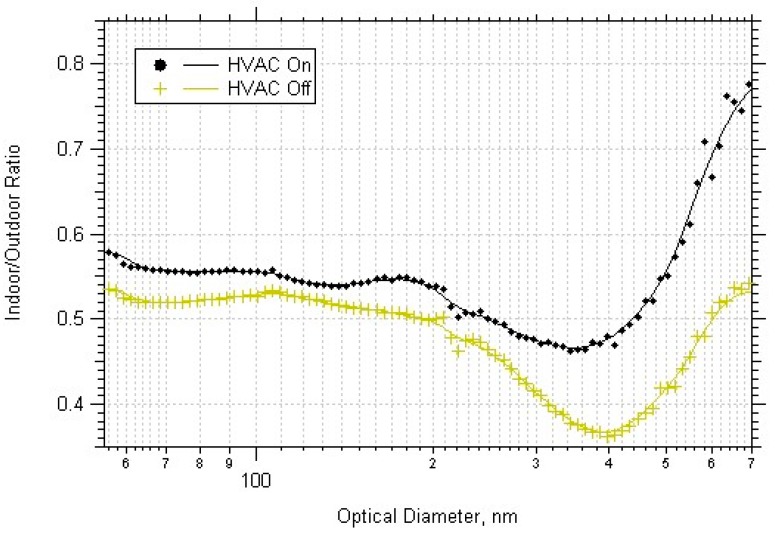
Indoor/outdoor ratios averaged over all datasets for the university building, separated by HVAC usage.

The indoor/outdoor ratio as a function of particle diameter varied for each building. At the Denver school, there was very little difference between the weekday and weekend, but higher I/O for day than night for all PM sizes. This result suggests that PM accumulated during the day due to occupant activities, and that the building was not occupied at all during the nighttime (occupancy supported by CO_2_ data, shown in Section D of Supplemental Data (Figures S31–S48). The office building I/O were highest during the weekend nights for all PM sizes. This suggests that the daytime HVAC usage decreased the amount of outdoor PM brought indoors, and that PM accumulated indoors when the HVAC was not on during the weekend nights. The Boulder school data had higher nighttime I/O for particle diameters < 200 nm. The weekday day I/O was lowest for all particles sizes except increased from 500–700 nm indicating particle resuspension from occupants. The university weekday I/O was higher than the weekend, and the nighttime was higher than the daytime, for diameters < 200 nm. Also, the weekday day and night I/O had a sharp increase for particles with diameters greater than 400 nm, possibly the effect of the particle resuspension due to the building occupants or cleaning activities. Figure 8 shows all the university data separated by times when the HVAC was on/off.

The university was the only place where the exact times of HVAC usage were obtained and thus the most accurate dataset regarding the effects of mechanical ventilation. This plot shows that HVAC usage in the university brought more PM into the building. The increase in the I/O at diameters closer to 1 µm may have been due to resuspension from building occupants or other various indoor activities, as indicated by the dramatic increase during weekday daytime periods for all of the buildings except the office building. The weekday nights can have some resuspension influence from as few as one person in the building near the sample location or from cleaning activities.

### 3.4. Indoor-Outdoor Regression Analysis

A regression analysis was performed between the indoor and outdoor PM concentrations measured with the UHSAS according to Ott et al. [30]. A least-squares linear fit was applied to each dataset to estimate the y-intercept. A lag time analysis was also performed to estimate the time it took for outdoor particles to appear indoors. After the lag was applied to the data the least-squares linear fit slope was estimated.

The y-intercept represents a baseline of indoor particle concentrations that exist regardless of outdoor particle concentrations. Each building’s usual indoor sources would show up in this baseline, as well as the equilibrium indoor concentration arising from infiltration. The regression analysis resulted in minimal y-intercepts (Table 4 and Table 5). Weekend and weekdays had similar behavior, with the highest y-intercepts during the night and for ultrafine (Table 4). The office building and Boulder school had the highest values occurring at night on the weekends for ultrafine PM (Table 5). Nights had higher y-intercept values than daytime suggesting that indoor sources were not the main contribution to baseline indoor concentrations but rather nighttime infiltration.

There was a time difference between when outdoor particles were measured and when indoor particles were measured: outdoor particles did not always immediately appear indoors. Some time lag is to be expected for particle transport, due to the building characteristics such as penetration factor, ventilation, and infiltration. The lag times for detection of the indoor air particles following that of outdoor were found using time series autocorrelation. A linear fit was applied to each dataset for both fine and ultrafine number concentrations, and the Pearson correlation coefficient was found. The lag times were applied to the data such that the outdoor event at time *t* corresponded to the indoor value at time *t +* ∆*t*, where ∆*t* is the lag time, a 12-min shift. The linear fit correlation coefficient was found again after the shift had been applied. This analysis was completed for each twelve-minute shift (the lag time resolution for this study is limited by the full switching cycle of twelve minutes) up to 600 min. The maximum correlation coefficient corresponds to the most probable lag time between indoor and outdoor PM concentrations.

Weekday days had the shortest average lag time of 11 min for ultrafine and fine PM (ranged from 8 to 24 min depending on the building). The lag time was shorter during the day than the night, and shorter during the weekdays than the weekends. Weekend nighttime lags ranged from 50 to 148 min. The nights had the biggest standard deviation in lag time, when the HVAC system was usually not operating, suggesting a large variability in the driving forces for infiltration.

The usage times of the HVAC system were only available at the university. Lag times were found to be 10–15 min for both ultrafine and fine PM when the HVAC system was in use, which agrees with the characteristic time due to air exchange of ¼ h = 15 min (Table 3). When the system was not in use, the lag time was 60–85 min and it was longer for ultrafine compared to fine particles. Section E of the Supplemental Data provides all lag time estimates (Tables S9–S12).

Once the appropriate lag time shifts were implemented into the data, the indoor number concentrations were plotted against those of outdoor and the least-squares linear fit was again applied to derive the slope. The slopes give an indication of how effectively outdoor PM travels into the indoor environments, regardless of outside levels. As the value approaches unity, the indoor air quality matches that of outdoor. The slope of the regression line provided an estimate of the dimensionless attenuation factor α= [p λ/(λ + k)], which depends on the physical building parameters penetration factor (p), the air exchange rate (λ), and the particle deposition rate (k). Far outliers were removed prior to the linear fit. Outliers were defined as three times the interquartile range above the 3rd quartile; results were not affected. The values of this correlation slope, like the lag times, are dependent on the buildings’ particular HVAC and ducting configuration, the seasonal effects, the period of the week, and the particle size. Table 6 details the slopes for ultrafine and fine PM for weekday daytime by season. Section C in the Supplemental Data provides all estimated slopes and correlation coefficients (Tables S1–S8).

To gain a better understanding of the effect of HVAC usage, the university building data was analyzed since the exact HVAC usage times were available. Table 7 shows the ultrafine particles entered the building more effectively than larger sized particles, and both particle sizes have higher correlation slopes when the HVAC system is in use.

The correlation coefficient is a measure of how well the linear fit to the indoor-outdoor data matches the actual data. The correlations were very good after the lag times were applied, ranging from 0.5 to 0.9 (data shown in Section C, Supplemental Data Tables S1, S3, S5 and S7). The ultrafine, on average, correlated better than the fine PM. The weekdays had higher correlation slopes than the weekends, but the day and night data were very comparable. The university data showed that when the HVAC system was not in use, the larger indoor particles had a lower correlation with outdoor values (0.65 compared to 0.79).

### 3.5. Ammonium Nitrate Infiltration

The Aerosol Mass Spectrometer data detailed the chemical speciation of sampled PM. Most of the PM sampled was organic, as has been reported previously [31,32]. Some trace amount of sulphates, ammonium, and nitrates were observed. There were some occasional events when nitrate fragments dominated the atmospheric PM, and the ammonium levels rose simultaneously with nitrates, suggesting that the makeup was ammonium nitrate particles. Figures of the mass concentration as a function of aerosol speciation for the outdoor samples are in Section F of the Supplemental Data (Figures S49–S56). For example, Figure S53 shows an elevated nitrate episode during winter at the Denver school.

Ammonium nitrate concentrations have been reported to be lower indoors than they would be based on penetration from outdoors and deposition losses alone due to transformation indoors of ammonium nitrate into nitric acid and ammonia gases from relative humidity and temperature differences [22,33]. To see if the data from this study agreed with this reduced indoor ammonium nitrate phenomena, the periods with high nitrate levels were extracted from the rest of their sample week. The two subsets of data were plotted as indoor against outdoor number concentrations, and the correlation was examined.

**Figure 9:**
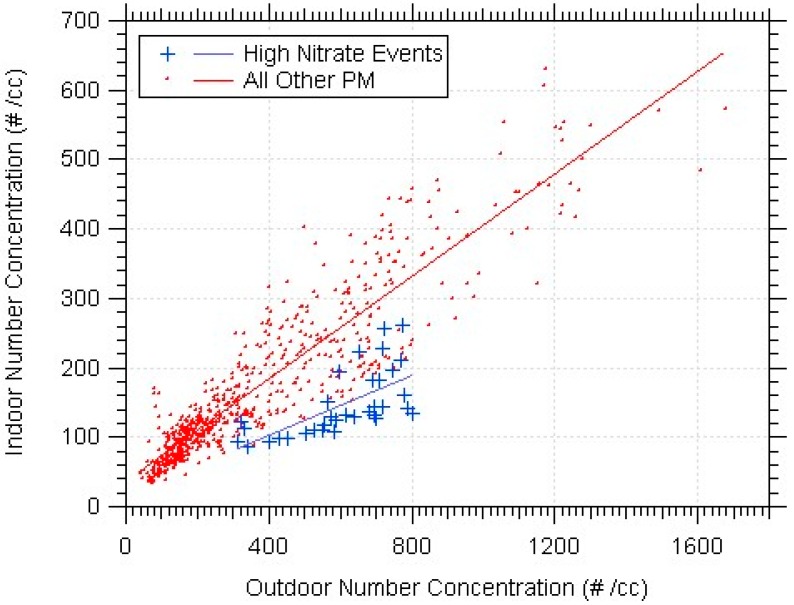
Indoor-outdoor fine PM correlation difference for a high ammonium nitrate event at the Denver school in the winter.

Figure 9 shows the indoor-outdoor fine number correlations at the Denver school during the winter (with lag times applied). The data during the ammonium nitrate event had a smaller linear-fit correlation slope, and the nitrate data fell below most of the other data on the curve. There were less indoor particles detected when the PM was composed of a fair amount of ammonium nitrate particles. There were only a few sampling weeks with a high ammonium nitrate event outdoors; each event corresponded to periods of reduced indoor particles.

## 4. Conclusions

Measurements in mechanically ventilated buildings were undertaken in this study to better understand the ventilation and infiltration of outdoor fine and ultrafine PM into typical indoor, commercial environments. The two Denver buildings had higher PM values than the two Boulder buildings, both outdoor and indoors. Additionally, the Denver average I/O ratio was higher than the ratio for the buildings in Boulder.

The average indoor/outdoor ratio ranged from 0.45 to 0.6 for all particle diameters from 55 to 700 nm, suggesting minimal particle removal as a function of size by the HVAC filters. The I/O ratio was positively correlated by the fresh air intake, but it was not a strong association. When the HVAC brought more fresh air into the building rather than recirculated air, the I/O ratio sometimes would increase. The ratio was not a strong function of building occupancy, when particle resuspension might be assumed. The only apparent particle resuspension that was observed were for the particles with diameters approaching 1 micron, but this was usually detectable only when the HVAC was not operating. This result suggests that the indoor PM concentrations were also influenced by infiltration into the building when the HVAC was not on (during the nighttime usually) and recirculation within the building.

A least-squares linear regression analysis showed that the background indoor number concentrations were minimal during the day and higher at night, suggesting very few indoor sources due to occupants. There was some lag time before high outdoor PM concentration events were seen indoors. When the HVAC system was operating, the lag time corresponded to the air exchange rate. There was an overall lag time of 42 min for ultrafine PM and 49 min for fine PM. The nighttime average lag time was only about one hour for both particle sizes suggesting fairly rapid infiltration into the building even in the absence of ventilation. When the HVAC system was not in use, the ultrafine particles had consistently shorter lag times than the fine PM. The regression analysis also showed that the ultrafine PM consistently had higher correlation slopes than the larger sized particles by nearly 20%. The slope depends on the penetration factor, the air exchange rate, and the particle deposition rate, and represents the effectiveness of a building shell and its ventilation filtration to protect indoor environments from outdoor PM. A higher slope indicates a less effective building shell and air filtration. Thus, the ultrafine particles are not removed as efficiently compared to the fine PM by either the building shell or filtration in the HVAC system.

The AMS allowed for chemical speciation of PM. Most of the time, the total PM mass was dominated by organics. There were a few events with high levels of ammonium nitrate outdoors. As shown in previous works [22,33], the ammonium nitrate seemed to infiltrate much less than predicted by penetration and deposition losses alone. When the ammonium nitrates dominated the outdoor mass concentration, the indoor-outdoor linear-fit slopes decreased. The indoor exposure to ammonium nitrate particles therefore, was minimal.

The results from this study show that fine PM indoors in mechanically ventilated buildings comes from outside. To reduce exposure, improved filtration is warranted, particularly for ultrafine particles. Nighttime infiltration for all particle sizes is significant depending on the building design and contributes to the total PM load in the building.

## Figures and Tables

**Table 1:** Description of heating, ventilation, and air-conditioning system and filters in each building.

Building Type	Location	Air-Handling Unit Type *	Supply Capacity (m^3^/h)	Filter Type	Rated Efficiency **	MERV ***
School	Denver	Variable Air Volume	54,400 (32,000 CFM)	10 cm (4 inch) pleated polyester pads	35%	NA
Office	Denver	Dual-duct	NA	5 cm (2 inch) pleated, pinch frame	30%–35%	8
School	Boulder	Constant Volume	28,000 (16,450 CFM)	5 cm (2 inch) pleated, high capacity	25%–30%	7
University	Boulder	Variable Air Volume	82,000 (48,300 CFM)	5 cm (2 inch) pleated, high capacity	30%–35%	8

* Variable air volume supplies variable airflow rates at constant temperature, constant volume supplies constant air flow at variable temperatures, dual-duct is a variable volume with a cold and hot air duct supplying the air; ** Rated Average Efficiency as described by ASHRAE standard 52.1 [22]; *** Minimum Efficiency Reporting Value (MERV) as described by ASHRAE standard 52.2 [18]. NA = not available.

**Table 2:** Summary of Testing Dates and Seasonal Average (SD) Temperatures (°C).

Building Type	Summer 2005	Fall 2005	Winter 2006	Spring 2006
School Denver	11–17 August	26 October–4 November	19–25 January	12–19 April
	17–22 August *			
Office Denver	22–29 August	30 November–5 December	25–30 January	19–25 April
School Boulder	29 August–6 September **	21–30 November	30 January–6 February	25 April–2 May
University Boulder	27 July–2 August	19–26 October	12–18 January	5–12 April
	3–10 August *			
Denver Temperature (°C)	21.6 (5.4)	6.9 (8.2)	1.1 (5.6)	13.2 (8.0)
Boulder Temperature (°C)	22.1 (5.6)	8.9 (5.9)	4.4 (4.3)	12.6 (6.1)

* The second sampling week was for UHSAS data, which does not overlap with the AMS data; ** The data from this sample week was flawed by a leak in the lines.

**Table 3:** Summary of air exchange and ventilation rates.

Sample Location	Air Exchange Rate (1/h)	Air Supply Airflow Rate (actual m^3^/h)	Interior Volume (m^3^)
School Denver	1.3–3.4	878	260
Office Denver	3.3–5.5	313	57
School Boulder (teacher’s lounge)	16–28	2450	88
School Boulder (music room during the day)	2.9–8.8	1800	204
School Boulder (music room at night)	0.114	-	204
University Boulder	4.0	34,700	8660

**Table 4:** Average least squares linear fit y-intercept values (SD) for weekday and weekend by particle size.

Y-Intercept (#/cm^3^)	Weekday		Weekend	
	Day	Night	Day	Night
Ultrafine	50 (5)	101 (8)	43 (11)	143 (7)
Fine	48 (2)	62 (2)	46 (5)	90 (3)

**Table 5:** Average least squares linear fit y-intercept values (Standard Deviation (SD)) for each building by time of day for ultrafine particles.

Y-Intercept (#/cm^3^)	University	School Denver	Office	School Boulder
Weekday Daytime	28 (5)	81 (8)	20 (9)	79 (5)
Weekend Nighttime	40 (11)	70 (10)	240 (10)	190 (10)

**Table 6:** Linear least-square fit slopes * to weekday daytime number concentration data by season.

Linear Least-Square Fit Slopes	Summer	Fall	Winter	Spring
Ultrafine	0.55	0.50	0.41	0.53
Fine	0.58	0.42	0.31	0.41
Fine, School Denver	0.78	0.52	0.30	0.64
Fine, Office	0.45	0.46	0.32	0.48
Fine, University	0.59	0.18	0.38	0.38
Fine, School Boulder	N/A	0.28	0.24	0.32

* estimated SD 0.01–0.03.

**Table 7:** Linear least-square fit slopes * to number concentration data by season.

Linear Least-Square Fit Slopes	HVAC System on	HVAC System off
Ultrafine	0.52	0.40
Fine	0.38	0.26

* estimated SD 0.01–0.02.

## Supplementary Materials

The supplementary materials are available online at www.mdpi.com/1660-4601/14/2/128/s1. Section A: Indoor and Outdoor UHSAS Number Concentration Plots. Figure S1. Summer 2005: University Building, Boulder, Figure S2. Summer 2005: Denver School, Figure S3. Summer 2005: Office building, Denver, Figure S4. Fall 2005: University Building, Boulder, Figure S5. Fall 2005: Denver School, Figure S6. Fall 2005: Boulder School, Figure S7. Fall 2005: Office building, Denver, Figure S8. Winter 2006: University Building, Boulder, Figure S9. Winter 2006: Denver School, Figure S10. Winter 2006: Office building, Denver, Figure S11. Winter 2006: Boulder School, Figure S12. Spring 2006: University Building, Boulder, Figure S13. Spring 2006: Denver School, Figure S14. Spring 2006: Office building, Denver, Figure S15. Spring 2006: Boulder School. Section B: Indoor and Outdoor PM_0.7_ Number Concentration Plots. Figure S16. Summer 2005: University Building, Boulder, Figure S17. Summer 2005: Denver School, Figure S18. Summer 2005: Office building, Denver, Figure S19. Fall 2005: University Building, Boulder, Figure S20. Fall 2005: Denver School, Figure S21. Fall 2005: Office building, Denver, Figure S22. Fall 2005: Boulder School, Figure S23. Winter 2006: University Building, Boulder, Figure S24. Winter 2006: Denver School, Figure S25. Winter 2006: Office building, Denver, Figure S26. Winter 2006: Boulder School, Figure S27. Spring 2006: University Building, Boulder, Figure S28. Spring 2006: Denver School, Figure S29. Spring 2006: Office building, Denver, Figure S30. Spring 2006: Boulder School. Section C: Tables of Average and Standard Deviations for Indoor/Outdoor Ratio, Slope, and Correlation Coefficient. Table S1. Combined yearlong indoor/outdoor ratio, slope, and correlation coefficient for each building, Table S2. Standard deviations of combined yearlong indoor/outdoor ratio and slope for each season, Table S3. Seasonal indoor/outdoor ratio, slope, and correlation coefficient, Table S4. Standard deviations for seasonal indoor/outdoor ratio and slope, Table S5. Indoor/outdoor ratio, slope, and correlation coefficient of entire dataset by period of the week, Table S6. Standard deviations for entire dataset for indoor/outdoor ratio and slope by periods of the week, Table S7. Seasonal indoor/outdoor ratio, slope, and correlation coefficient for University building only, by HVAC usage, Table S8. Standard deviations for indoor/outdoor ratio and slope for University building only, by HVAC usage. Section D: Indoor and Outdoor CO_2_ Concentration Plots. Figure S31. Summer 2005: University Building, Boulder (week 1, coincides with UHSAS data), Figure S32. Summer 2005: University Building, Boulder (week 2, coincides with AMS data), Figure S33. Summer 2005: Denver School (week 1, coincides with AMS), Figure S34. Summer 2005: Denver School (week 2, coincides with UHSAS), Figure S35. Summer 2005: Office building, Denver, Figure S36. Summer 2005: Boulder School, Figure S37. Fall 2005: University Building, Boulder, Figure S38. Fall 2005: Denver School, Figure S39. Fall 2005: Boulder School, Figure S40. Fall 2005: Office building, Denver, Figure S41. Winter 2006: University Building, Boulder, Figure S42. Winter 2006: Denver School, Figure S43. Winter 2006: Office building, Denver, Figure S44. Winter 2006: Boulder School, Figure S45. Spring 2006: University Building, Boulder, Figure S46. Spring 2006: Denver School, Figure S47. Spring 2006: Office building, Denver, Figure S48. Spring 2006: Boulder School. Section E: Tables of Average Lag Times and Standard Deviations. Table S9. Average (SD) lag times for each building, Table S10. Average (SD) lag times for each season, Table S11. Average (SD) lag times for period of the week, Table S12. Seasonal Average (SD) lag times for university building only, by HVAC usage. Section F: Outdoor Hour-Averaged AMS Chemical Data Plots, Figure S49. Summer 2005: Denver School, Figure S50. Summer 2005: University Building, Boulder, Figure S51. Fall 2005: Denver School, Figure S52. Fall 2005: University Building, Boulder, Figure S53. Winter 2006: Denver School, Figure S54. Winter 2006: University Building, Boulder, Figure S55. Spring 2006: Denver School, Figure S56. Spring 2006: University Building, Boulder.
