# Supplementary material for: Ultrafine and Fine Particulate Matter Inside and Outside of Mechanically Ventilated Buildings
Source: Int J Environ Res Public Health. 2017 Jan 28;14(2):128. doi: 10.3390/ijerph14020128 (PMC5334682; doi:10.3390/ijerph14020128)
Supplement: Supplementary file 1 [file ijerph-14-00128-s001.pdf]

Supplementary Materials: Ultrafine and Fine Particulate Matter inside and outside of Mechanically Ventilated Buildings

Shelly L. Miller, Nick A. Facciola, Darin Toohey and John Zhai

1. Section A: Indoor and Outdoor UHSAS Number Concentration Plots

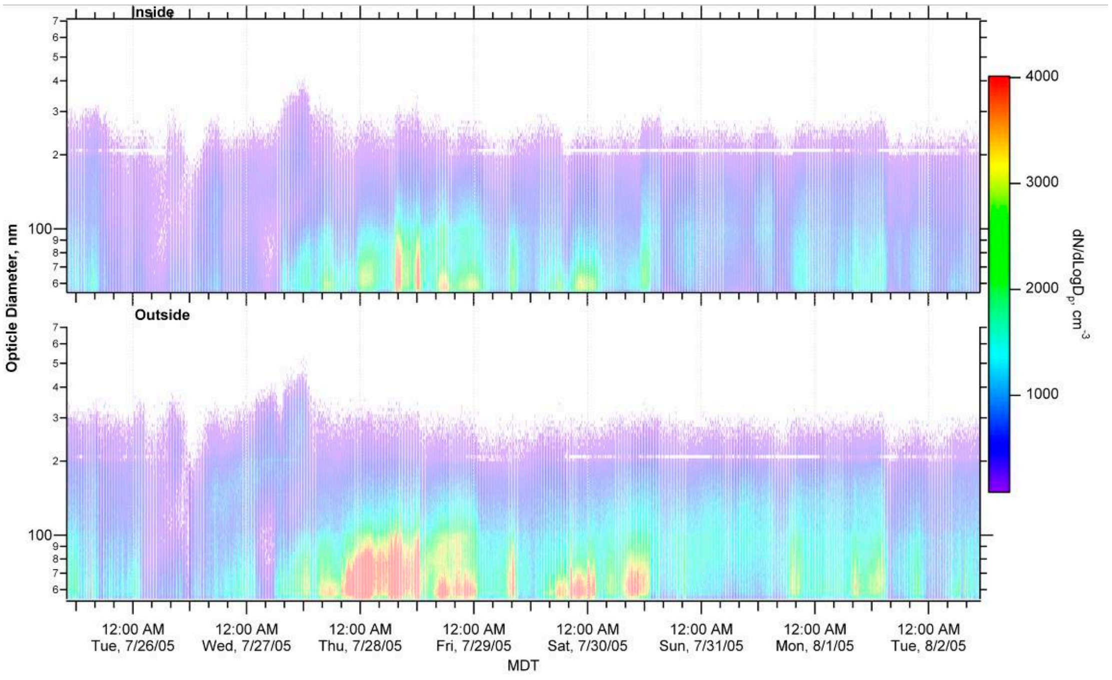

Figure S1. Summer 2005: University Building, Boulder.

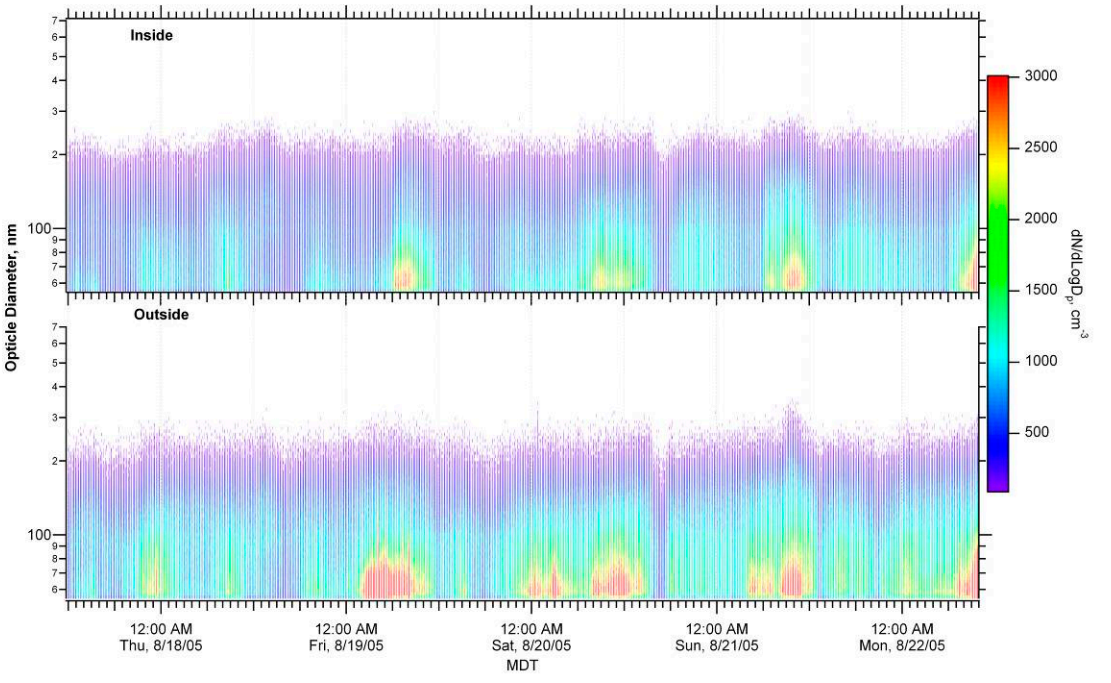

Figure S2. Summer 2005: Denver School.

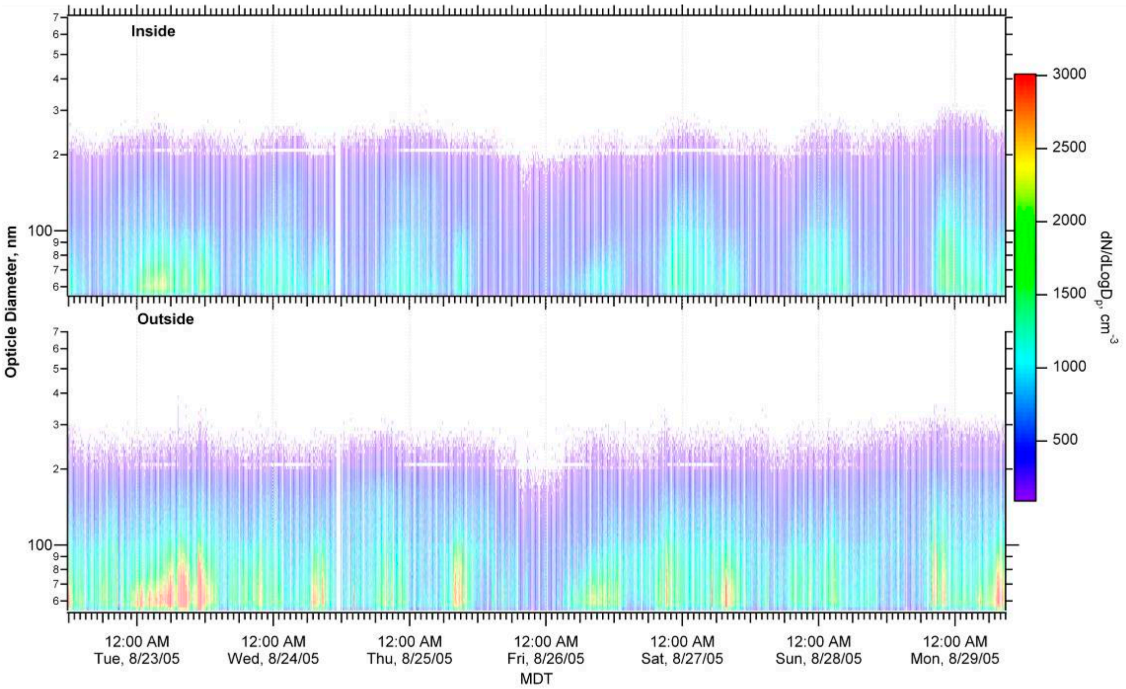

Figure S3. Summer 2005: Office building, Denver.

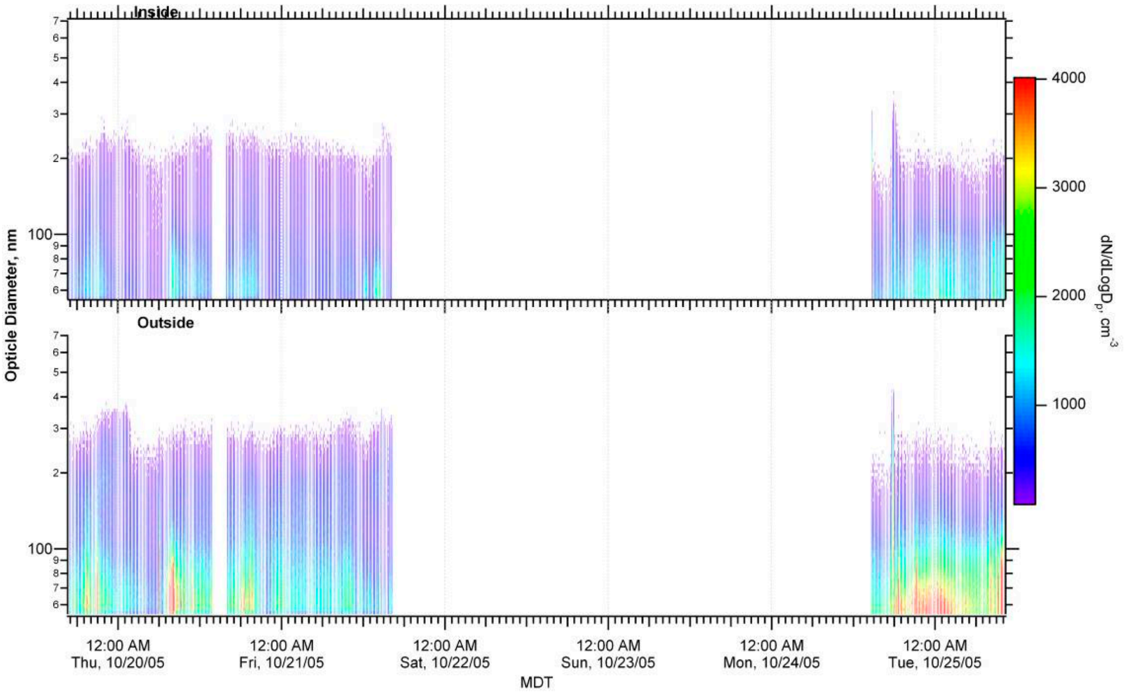

Figure S4. Fall 2005: University Building, Boulder.

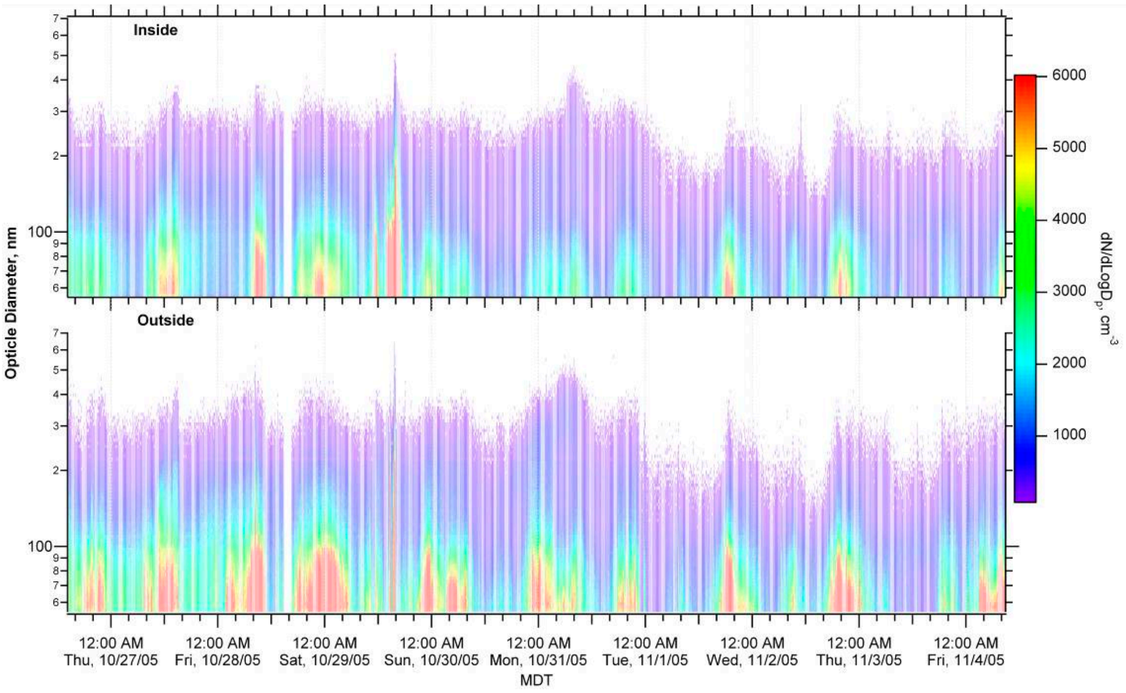

Figure S5. Fall 2005: Denver School.

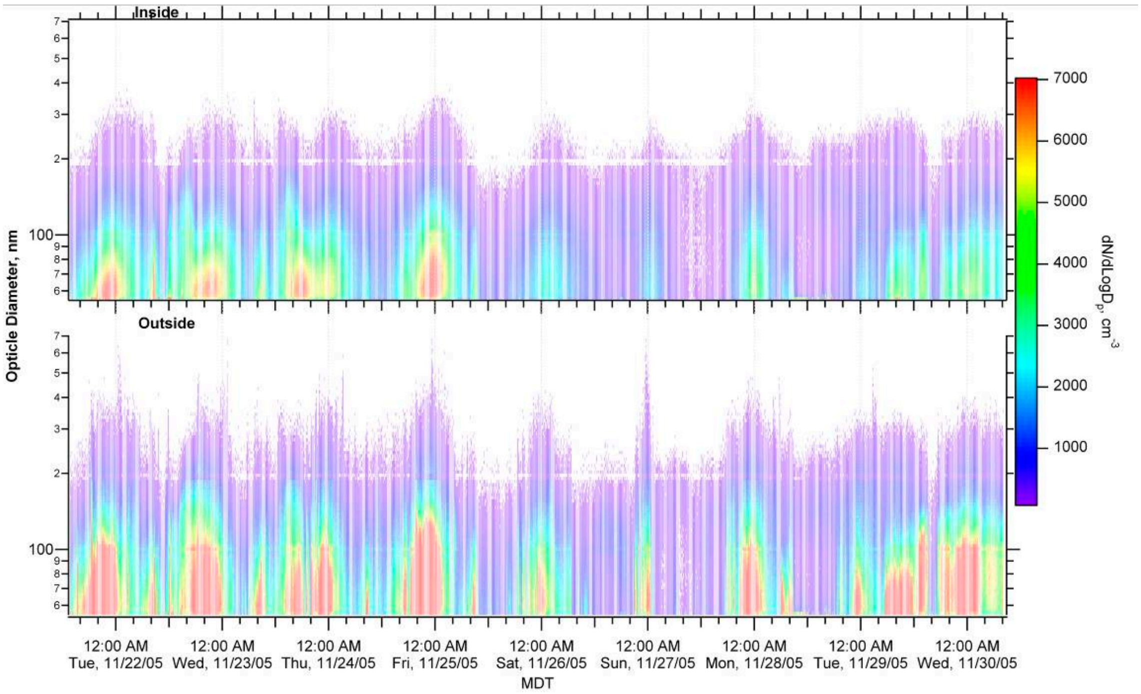

Figure S6. Fall 2005: Boulder School.

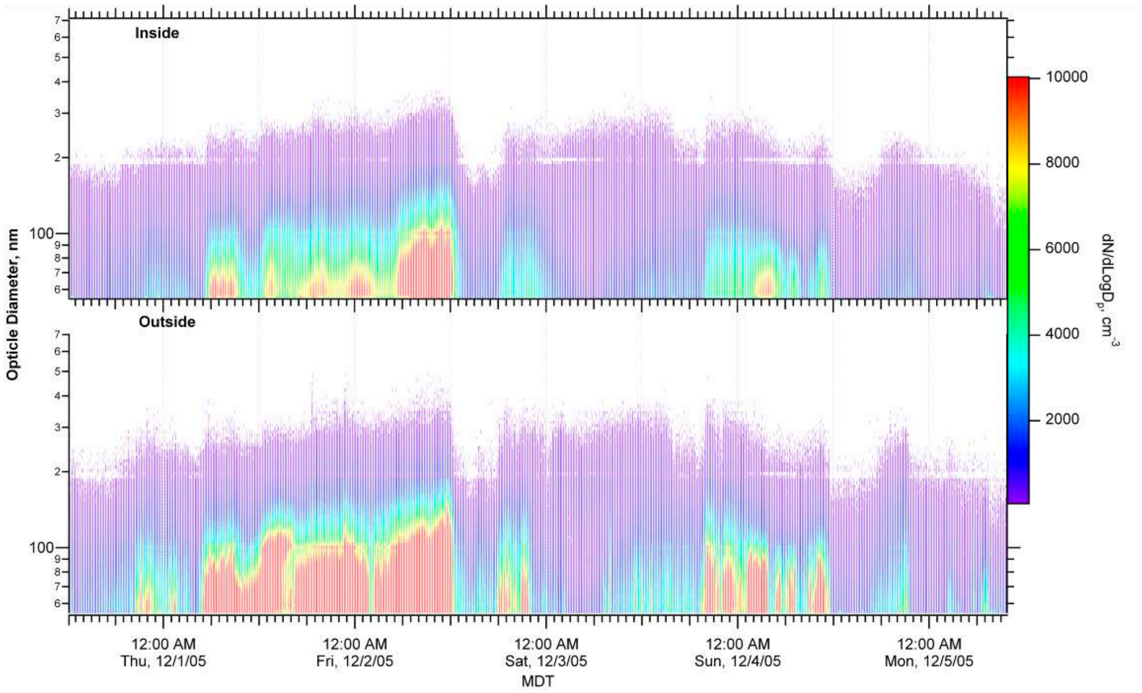

Figure S7. Fall 2005: Office building, Denver.

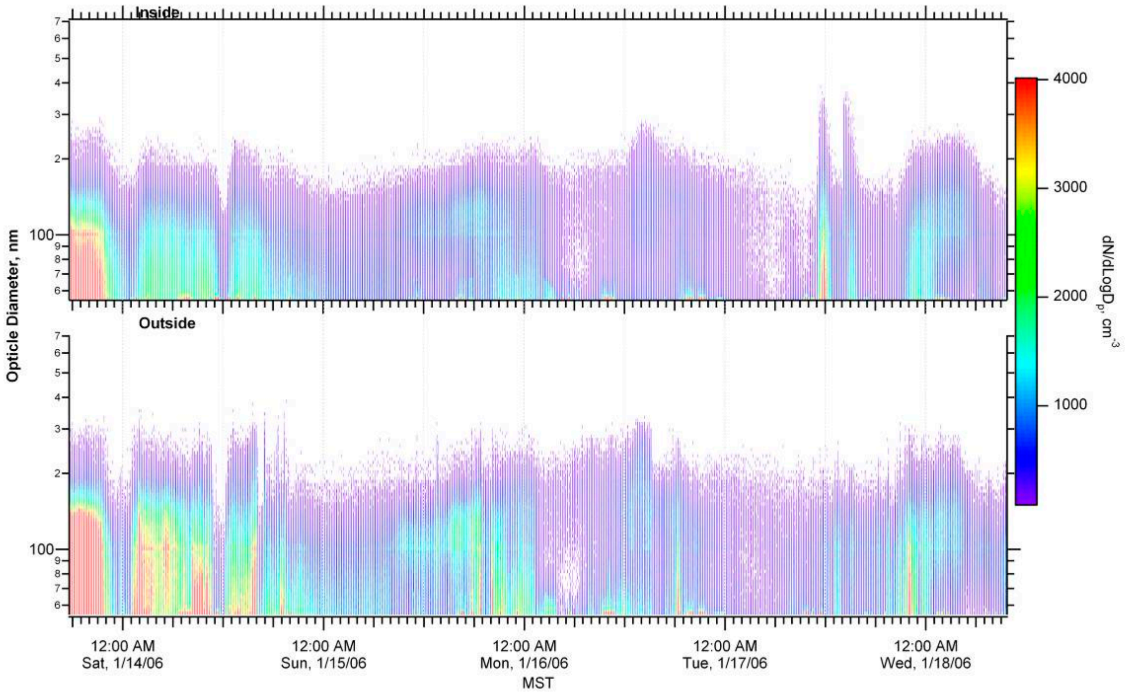

Figure S8. Winter 2006: University Building, Boulder.

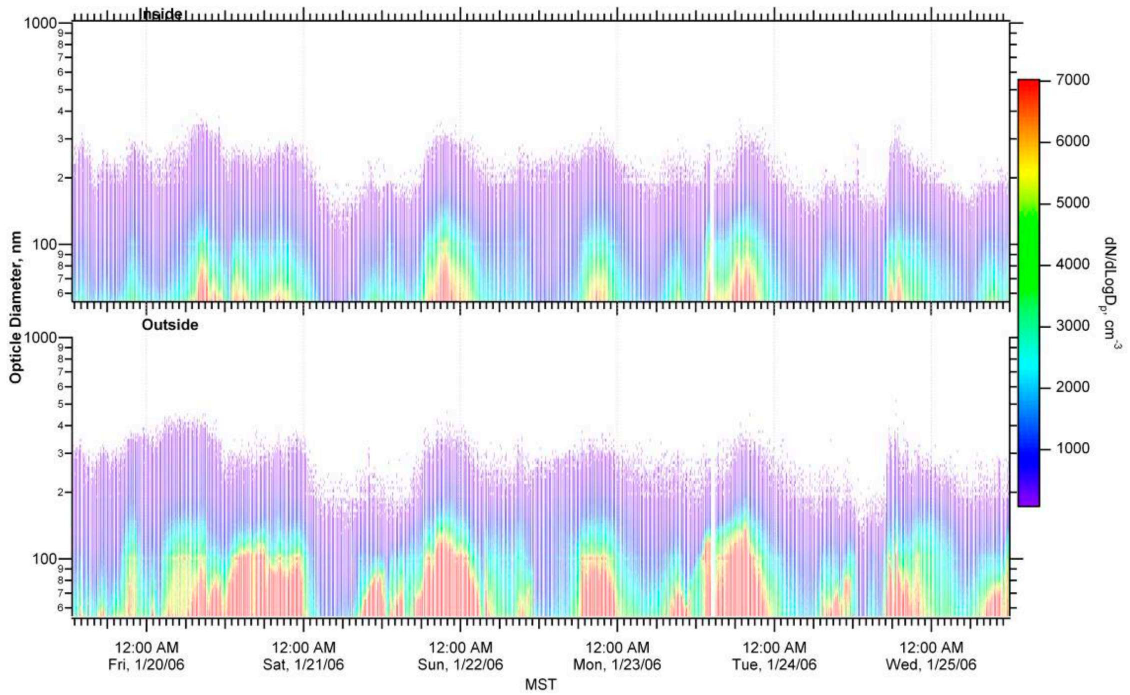

Figure S9. Winter 2006: Denver School.

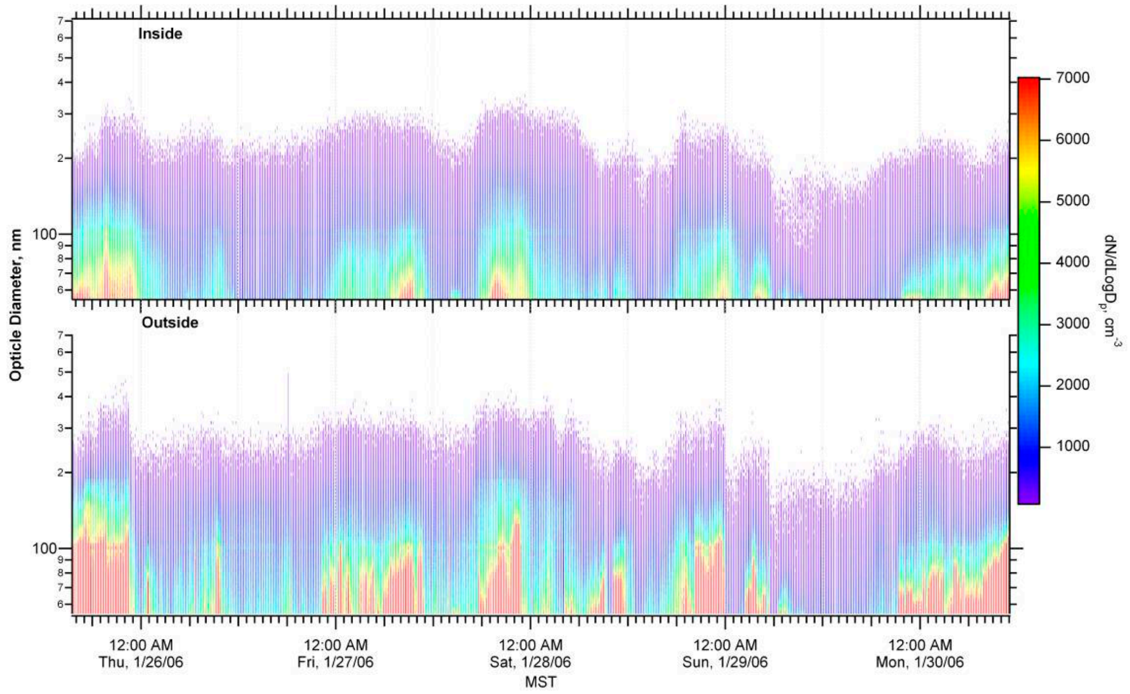

Figure S10. Winter 2006: Office building, Denver.

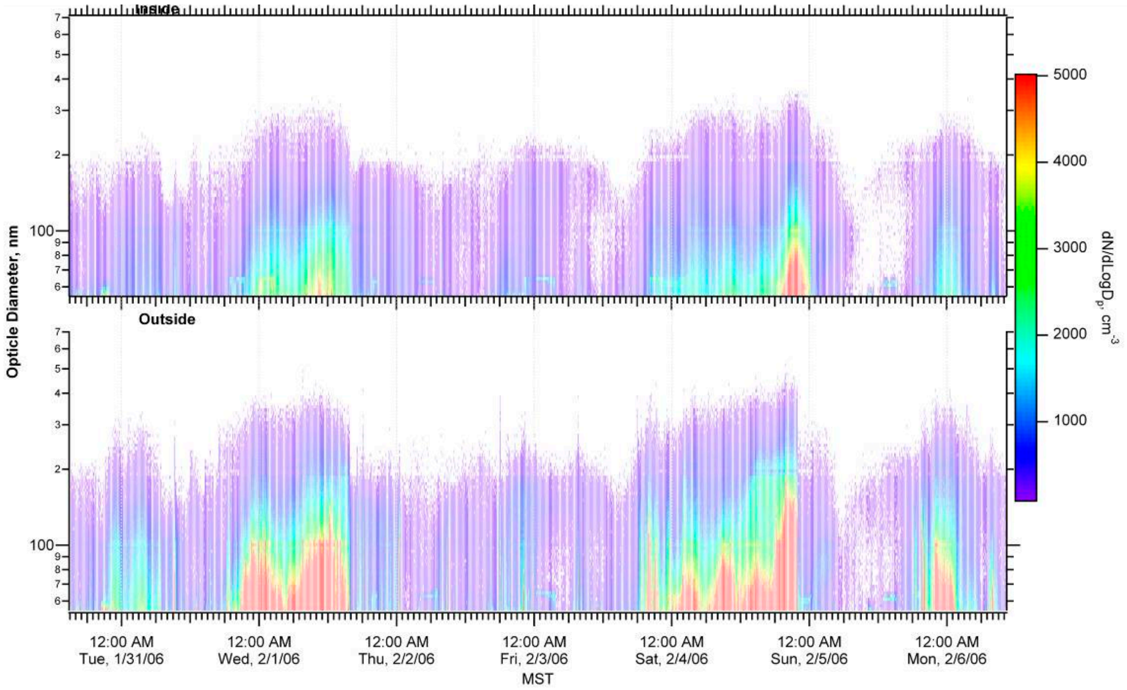

Figure S11. Winter 2006: Boulder School.

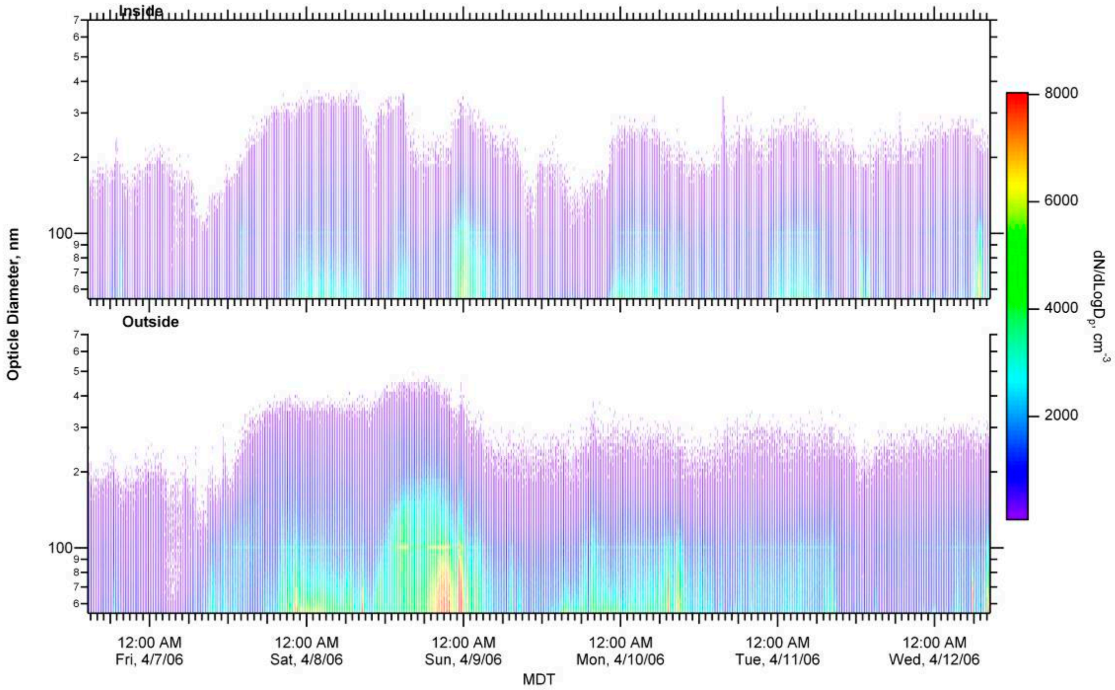

Figure S12. Spring 2006: University Building, Boulder.

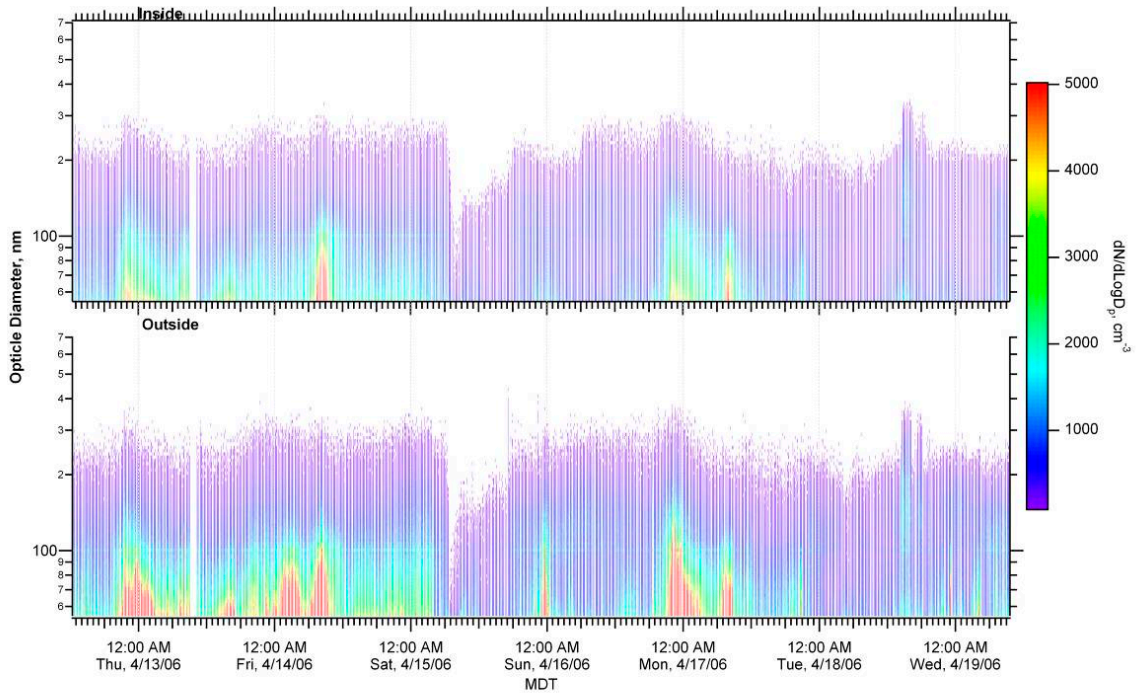

Figure S13. Spring 2006: Denver School.

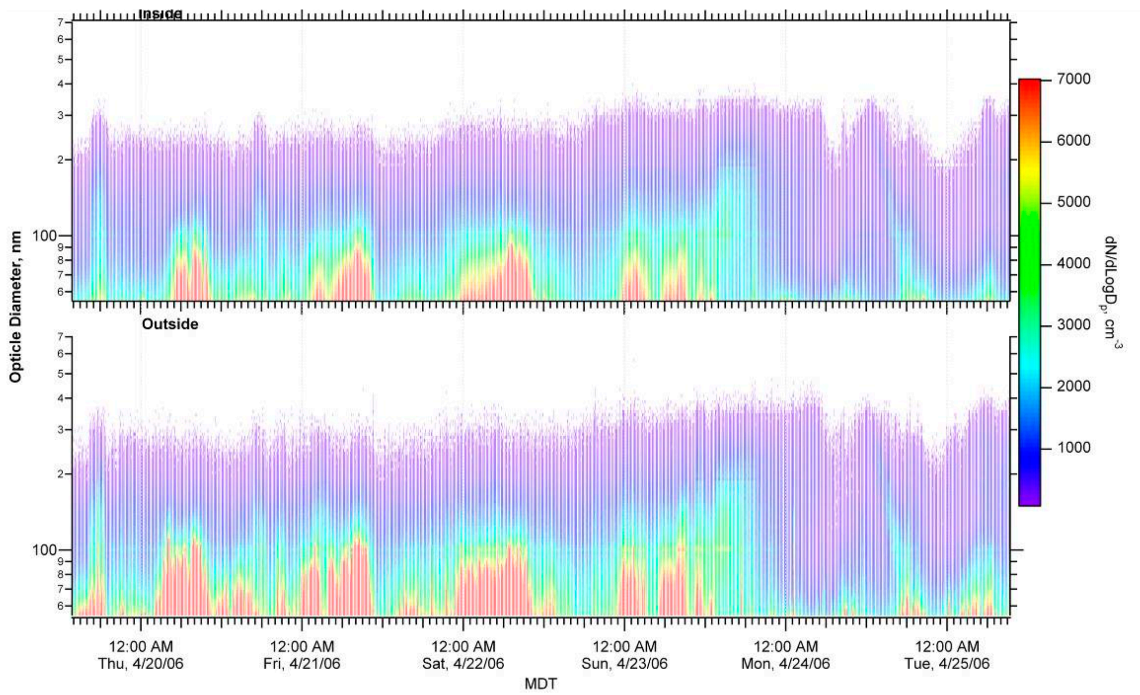

Figure S14. Spring 2006: Office building, Denver.

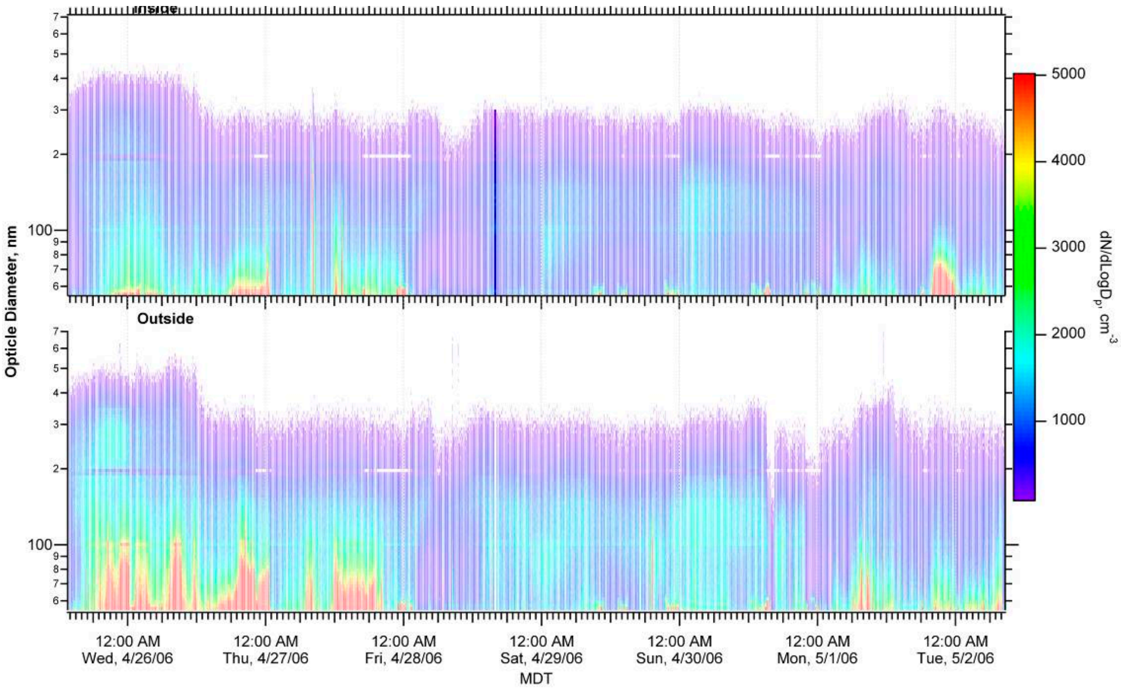

Figure S15. Spring 2006: Boulder School.

2. Section B: Indoor and Outdoor PM0.7 Number Concentration Plots

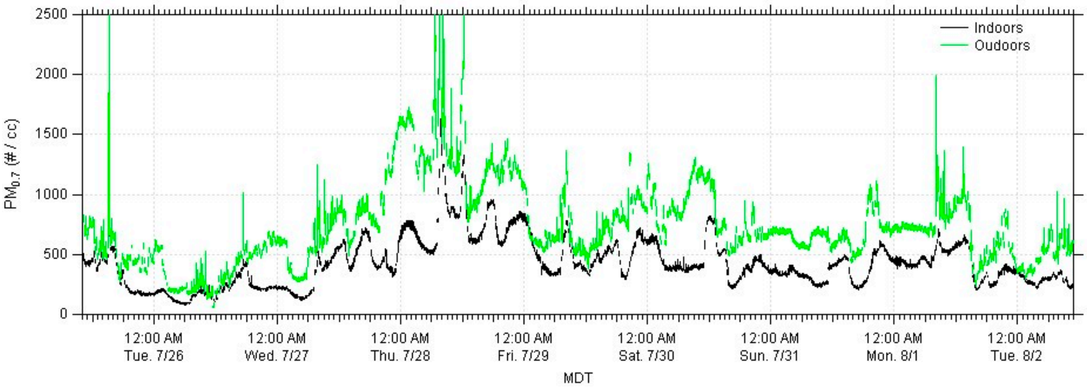

Figure S16. Summer 2005: University Building, Boulder.

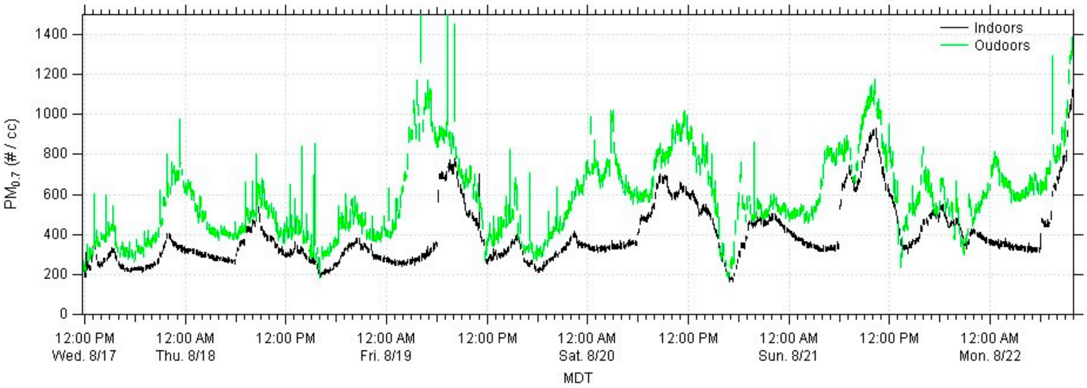

Figure S17. Summer 2005: Denver School.

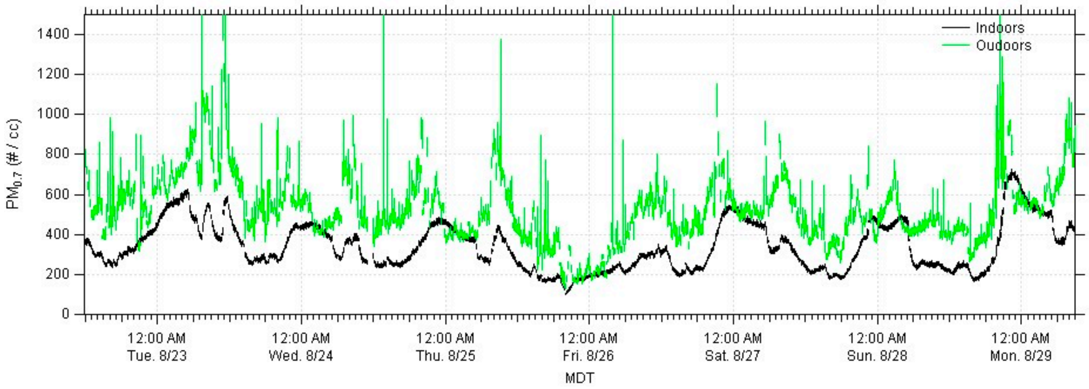

Figure S18. Summer 2005: Office building, Denver.

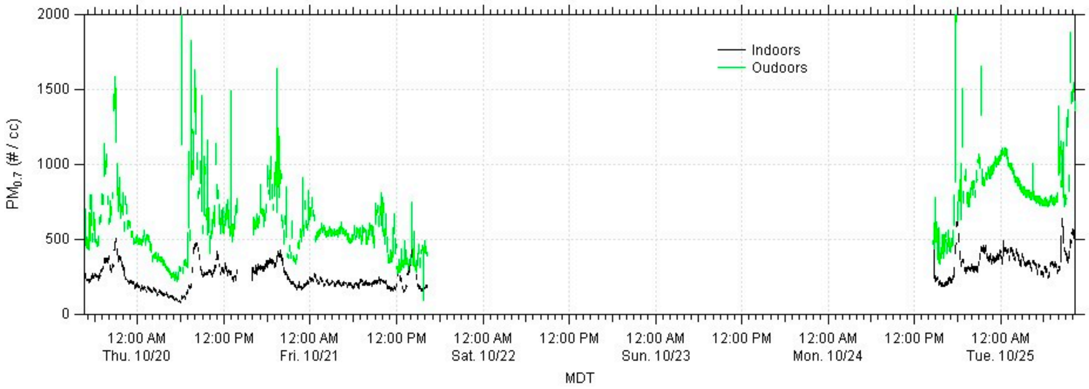

Figure S19. Fall 2005: University Building, Boulder.

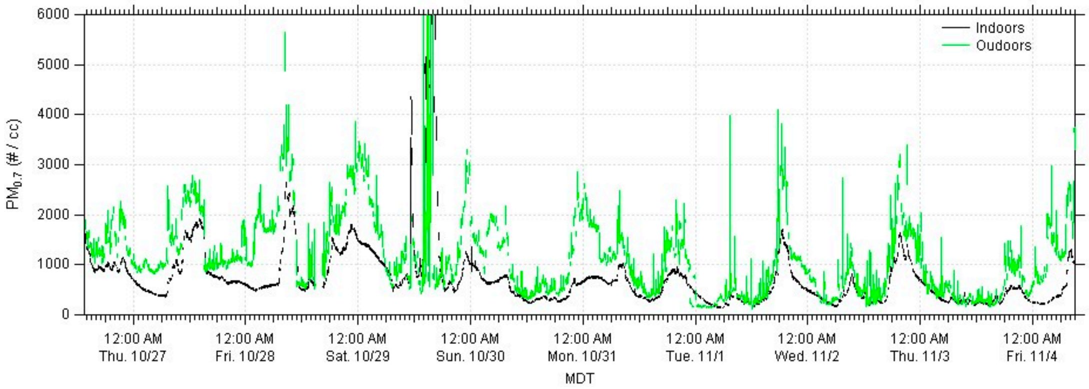

Figure S20. Fall 2005: Denver School.

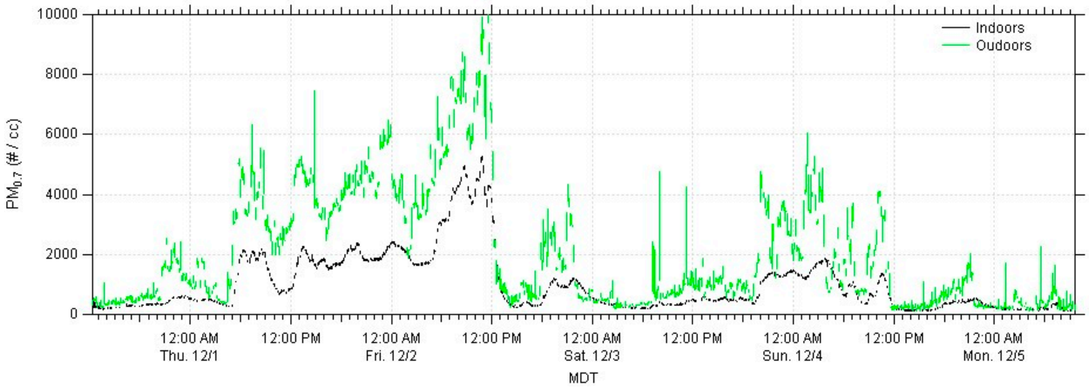

Figure S21. Fall 2005: Office building, Denver.

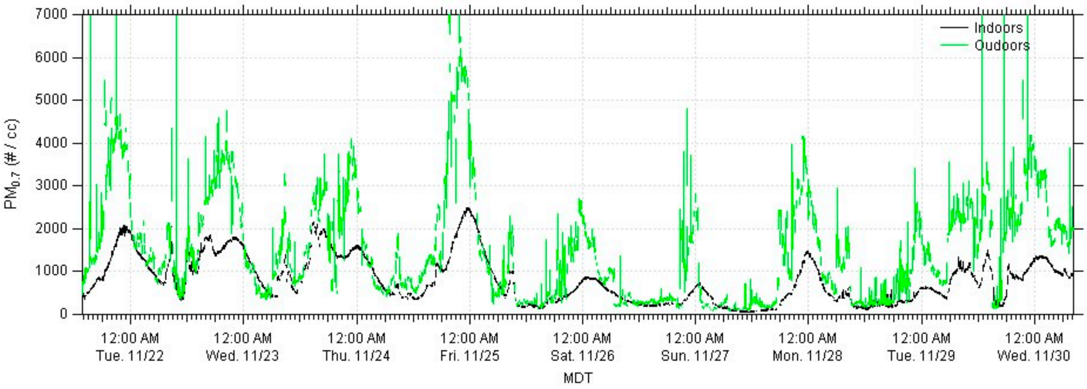

Figure S22. Fall 2005: Boulder School.

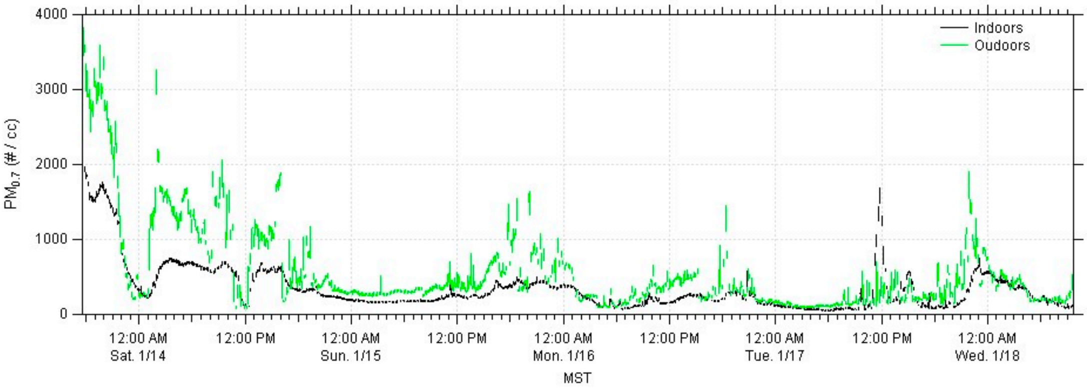

Figure S23. Winter 2006: University Building, Boulder.

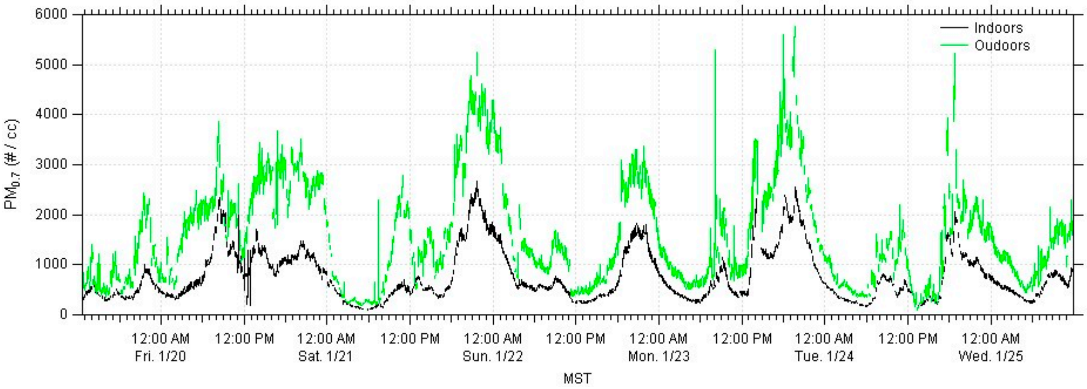

Figure S24. Winter 2006: Denver School.

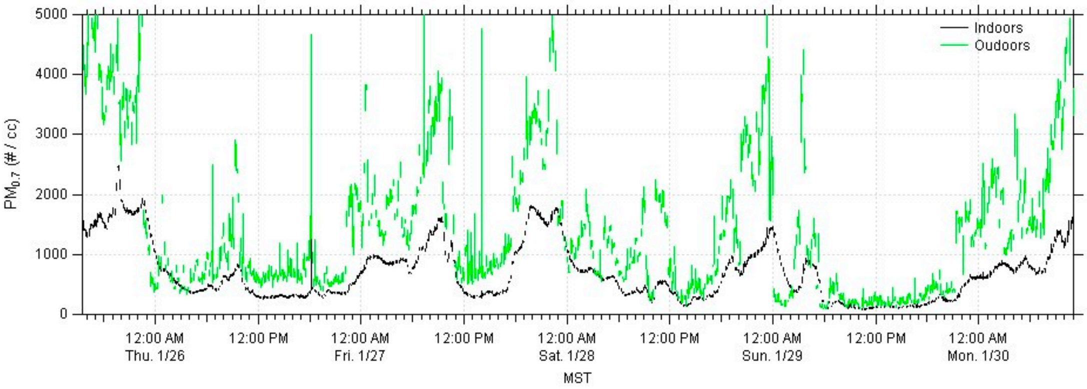

Figure S25. Winter 2006: Office building, Denver.

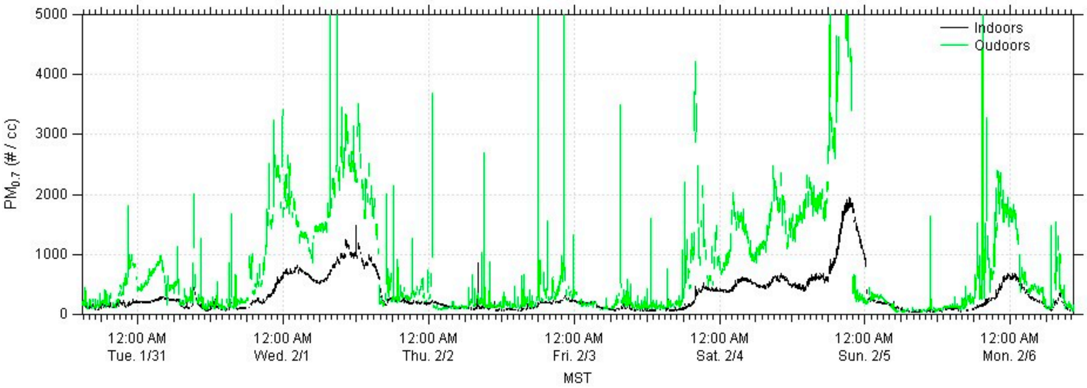

Figure S26. Winter 2006: Boulder School.

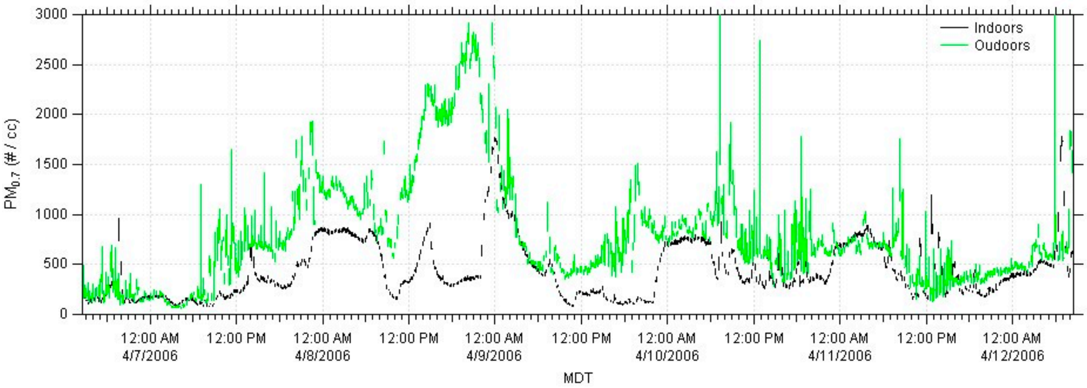

Figure S27. Spring 2006: University Building, Boulder.

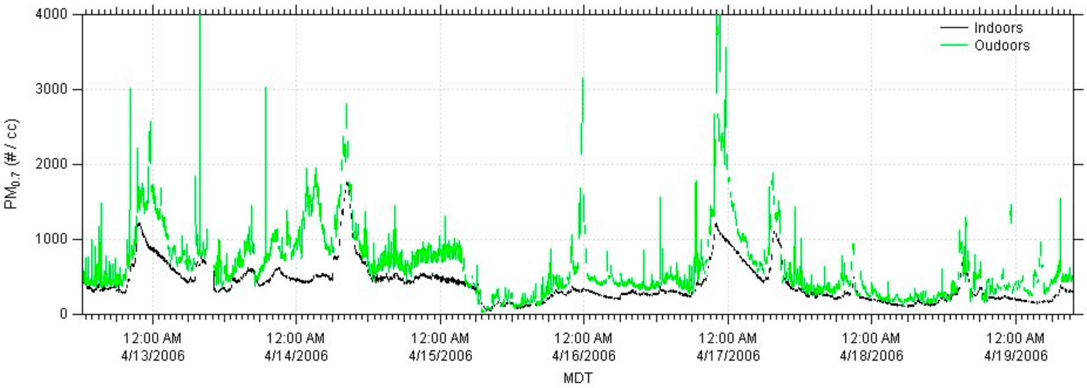

Figure S28. Spring 2006: Denver School.

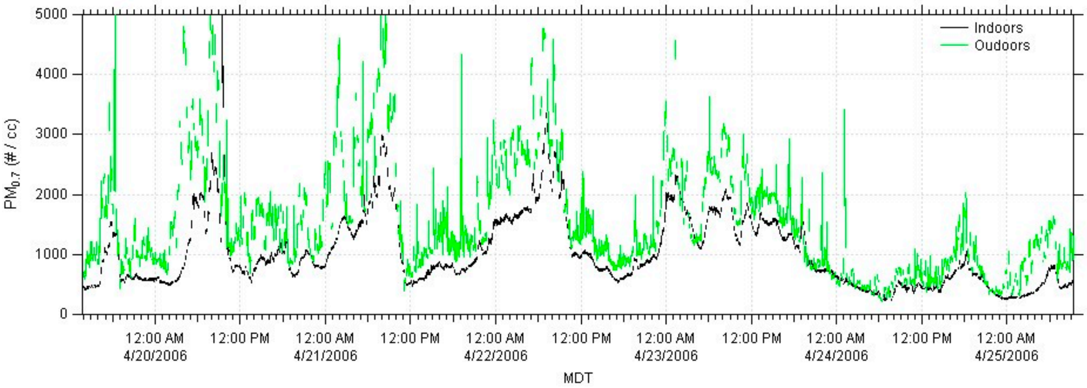

Figure S29. Spring 2006: Office building, Denver.

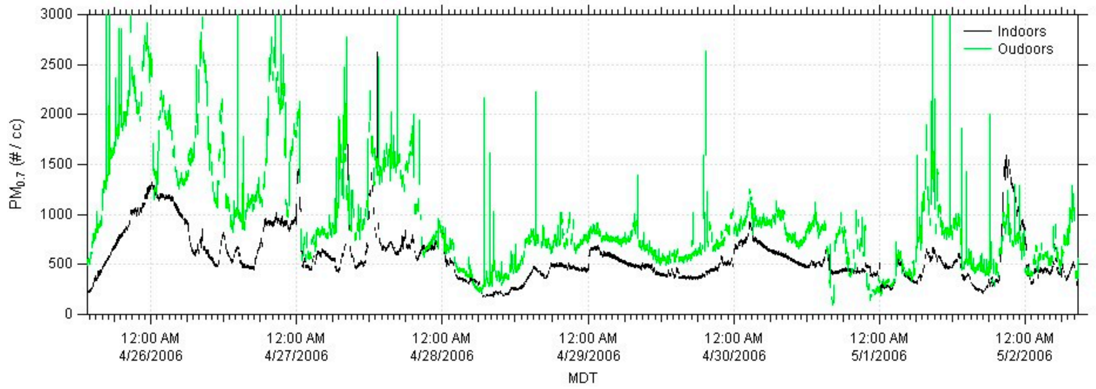

Figure S30. Spring 2006: Boulder School.

3. Section C: Tables of Average and Standard Deviations for Indoor/Outdoor Ratio, Slope, and Correlation Coefficient

Table S1. Combined yearlong indoor/outdoor ratio, slope, and correlation coefficient for each building.

Building Type	Day Type	Time of Day	Ratio		Slope		r_p	
			Ultrafine	Fine	Ultrafine	Fine	Ultrafine	Fine
University		All	0.617	0.581	0.451	0.279	0.806	0.656
	Weekday	Day	0.621	0.588	0.512	0.410	0.834	0.809
		Night	0.678	0.615	0.451	0.416	0.817	0.768
	Weekend	Day	0.518	0.521	0.260	0.073	0.626	0.270
		Night	0.573	0.548	0.474	0.332	0.875	0.782
Denver School		All	0.644	0.670	0.457	0.413	0.770	0.799
	Weekday	Day	0.654	0.707	0.478	0.396	0.892	0.851
		Night	0.597	0.604	0.420	0.341	0.834	0.776
	Weekend	Day	0.757	0.763	0.527	0.466	0.526	0.772
		Night	0.565	0.572	0.430	0.394	0.936	0.925
Office		All	0.632	0.642	0.457	0.428	0.903	0.867
	Weekday	Day	0.548	0.579	0.499	0.418	0.942	0.918
		Night	0.678	0.686	0.378	0.299	0.879	0.854
	Weekend	Day	0.572	0.620	0.570	0.744	0.894	0.962
		Night	0.840	0.748	0.359	0.413	0.813	0.744
Boulder School		All	0.676	0.635	0.379	0.310	0.818	0.770
	Weekday	Day	0.583	0.569	0.393	0.322	0.813	0.798
		Night	0.738	0.638	0.396	0.346	0.835	0.842
	Weekend	Day	0.612	0.642	0.222	0.189	0.861	0.543
		Night	0.948	0.873	0.264	0.211	0.735	0.623

All = All data from that building; Day = 6 am–8 pm; Night = 8 pm–6 am.

Table S2. Standard deviations of combined yearlong indoor/outdoor ratio and slope for each season.

Building Type	Day Type	Time of Day	Ratio Standard Deviation		Slope Standard Deviation	
			Ultrafine	Fine	Ultrafine	Fine
University		All	0.3502	0.2263	0.0069	0.0067
	Weekday	Day	0.2972	0.1800	0.0112	0.0098
		Night	0.4381	0.2255	0.0117	0.0127
	Weekend	Day	0.3414	0.3245	0.0169	0.0135
		Night	0.1734	0.1773	0.0162	0.0165
Denver School		All	0.4091	0.3105	0.0070	0.0058

	Weekday	Day	0.1851	0.1946	0.0070	0.0071
		Night	0.3455	0.2353	0.0094	0.0094
	Weekend	Day	0.7952	0.5712	0.0377	0.0170
		Night	0.1776	0.1405	0.0087	0.0088
Office		All	0.3335	0.1987	0.0042	0.0048
	Weekday	Day	0.1773	0.1443	0.0057	0.0058
		Night	0.2943	0.2199	0.0076	0.0067
	Weekend	Day	0.1738	0.1231	0.0122	0.0090
		Night	0.6375	0.2810	0.0132	0.0190
Boulder School		All	0.5540	0.4095	0.0052	0.0050
	Weekday	Day	0.3311	0.2253	0.0085	0.0073
		Night	0.6473	0.3409	0.0091	0.0078
	Weekend	Day	0.3291	0.5159	0.0065	0.0145
		Night	0.9543	0.7410	0.0144	0.0157

All = All data from that building; Day = 6 am–8 pm; Night = 8 pm–6 am.

Table S3. Seasonal indoor/outdoor ratio, slope, and correlation coefficient.

Season	Day Type	Time of Day	Ratio		Slope		rp	
			Ultrafine	Fine	Ultrafine	Fine	Ultrafine	Fine
Summer	Weekday	Day	0.549	0.575	0.625	0.663	0.892	0.848
		Night	0.373	0.377	0.662	0.667	0.778	0.601
	Weekend	Day	0.609	0.528	0.596	0.640	0.826	0.678
		Night	0.291	0.249	0.693	0.722	0.536	0.414
Fall	Weekday	Day	0.495	0.411	0.621	0.616	0.912	0.874
		Night	0.401	0.331	0.630	0.590	0.856	0.863
	Weekend	Day	0.564	0.472	0.767	0.713	0.537	0.772
		Night	0.318	0.262	0.797	0.649	0.821	0.795
Winter	Weekday	Day	0.406	0.308	0.539	0.526	0.906	0.892
		Night	0.400	0.311	0.687	0.578	0.860	0.835
	Weekend	Day	0.278	0.178	0.534	0.550	0.850	0.722
		Night	0.358	0.291	0.764	0.686	0.860	0.837
Spring	Weekday	Day	0.534	0.405	0.619	0.650	0.904	0.871
		Night	0.364	0.483	0.715	0.718	0.796	0.901
	Weekend	Day	0.670	0.447	0.625	0.700	0.927	0.687
		Night	0.528	0.523	0.681	0.700	0.873	0.748

Day = 6 am–8 pm; Night = 8 pm–6 am.

Table S4. Standard deviations for seasonal indoor/outdoor ratio and slope.

Season	Day Type	Time of Day	Ratio Standard Deviation		Slope Standard Deviation	
			Ultrafine	Fine	Ultrafine	Fine
Summer	Weekday	Day	0.1651	0.1302	0.0091	0.0117
		Night	0.3322	0.1929	0.0117	0.0196
	Weekend	Day	0.1631	0.1526	0.0207	0.0283
		Night	0.1982	0.1606	0.0285	0.0349
Fall	Weekday	Day	0.3144	0.2439	0.0062	0.0064
		Night	0.4633	0.3238	0.0077	0.0062
	Weekend	Day	0.9130	0.5492	0.0449	0.0195
		Night	0.8226	0.3421	0.0131	0.0118
Winter	Weekday	Day	0.2475	0.1816	0.0063	0.0051
		Night	0.6382	0.2637	0.0088	0.0075
	Weekend	Day	0.3192	0.3117	0.0073	0.0073

		Night	0.7741	0.6333	0.0109	0.0097
Spring	Weekday	Day	0.2537	0.1760	0.0079	0.0072
		Night	0.3051	0.1929	0.0099	0.0083
	Weekend	Day	0.2664	0.5476	0.0123	0.0213
		Night	0.2939	0.2808	0.0159	0.0251

Day = 6 am–8pm; Night = 8 pm–6 am.

Table S5. Indoor/outdoor ratio, slope, and correlation coefficient of entire dataset by period of the week.

Day Type	Time of Day	Ratio		Slope		r_p	
		Ultrafine	Fine	Ultrafine	Fine	Ultrafine	Fine
	All	0.643	0.635	0.442	0.374	0.854	0.800
Weekday	Day	0.603	0.615	0.476	0.382	0.909	0.861
	Night	0.671	0.634	0.404	0.349	0.863	0.841
Weekend	Day	0.622	0.644	0.477	0.409	0.681	0.713
	Night	0.734	0.689	0.379	0.320	0.846	0.741
Weekday	Day	0.632	0.623	0.448	0.369	0.891	0.853
	Night	0.668	0.662	0.424	0.382	0.753	0.720
Weekend	Day	0.609	0.624	0.477	0.391	0.856	0.795
	Night	0.689	0.650	0.397	0.343	0.858	0.812

All = All data from entire dataset; Day = 6 am–8 pm; Night = 8 pm–6 am.

Table S6. Standard deviations for entire dataset for indoor/outdoor ratio and slope by periods of the week.

Day Type	Time of Day	Ratio Standard Deviation		Slope Standard Deviation	
		Ultrafine	Fine	Ultrafine	Fine
	All	0.4226	0.3015	0.0026	0.0027
Weekday	Day	0.2589	0.1989	0.0034	0.0035
	Night	0.4565	0.2631	0.0042	0.0040
Weekend	Day	0.4897	0.4270	0.0120	0.0093
	Night	0.6045	0.4199	0.0067	0.0082
Weekday	Day	0.3594	0.2290	0.0027	0.0026
	Night	0.5426	0.4246	0.0066	0.0066
Weekend	Day	0.3462	0.2892	0.0037	0.0038
	Night	0.5043	0.3169	0.0036	0.0037

All = All data from entire dataset; Day = 6 am–8 pm; Night = 8 pm–6 am.

Table S7. Seasonal indoor/outdoor ratio, slope, and correlation coefficient for University building only, by HVAC usage.

Season	HVAC Usage	Ratio		Slope		r_p	
		Ultrafine	Fine	Ultrafine	Fine	Ultrafine	Fine
Summer	on	0.642	0.648	0.539	0.600	-	-
	off	0.555	0.542	0.420	0.427	-	-
Fall	on	0.478	0.440	0.238	0.205	-	-
	off	0.375	0.417	0.354	0.319	-	-
Winter	on	0.644	0.561	0.621	0.365	-	-
	off	0.788	0.666	0.380	0.269	-	-
Spring	on	0.617	0.571	0.282	0.222	-	-
	off	0.616	0.570	0.382	0.193	-	-
Total	on	0.610	0.581	0.514	0.364	0.837	0.788

	off	0.612	0.568	0.407	0.259	0.778	0.653

Total = all seasons combined.

Table S8. Standard deviations for indoor/outdoor ratio and slope for University building only, by HVAC usage.

Season	HVAC Usage	Ratio Standard Deviation		Slope Standard Deviation	
		Ultrafine	Fine	Ultrafine	Fine
Summer	on	0.183	0.128	0.012	0.0171
	off	0.183	0.132	0.012	0.0207
Fall	on	0.246	0.122	0.0147	0.0129
	off	0.122	0.043	0.017	0.0148
Winter	on	0.472	0.322	0.0125	0.0069
	off	0.643	0.320	0.014	0.0134
Spring	on	0.410	0.236	0.0359	0.0142
	off	0.334	0.297	0.0279	0.0189
Total	on	0.323	0.213	0.010	0.008
	off	0.410	0.245	0.010	0.009

Total = all seasons combined.

4. Section D: Indoor and Outdoor CO_2 Concentration Plots

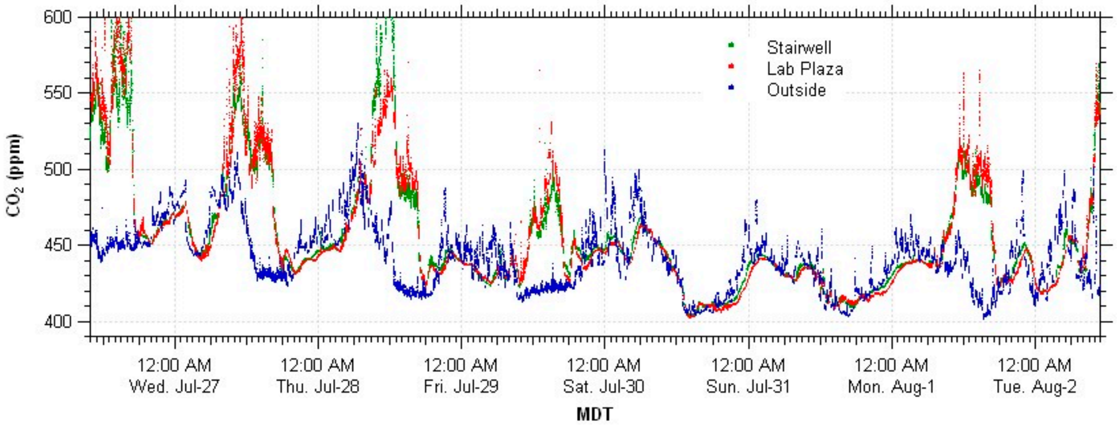

Figure S31. Summer 2005: University Building, Boulder (week 1, coincides with UHSAS data).

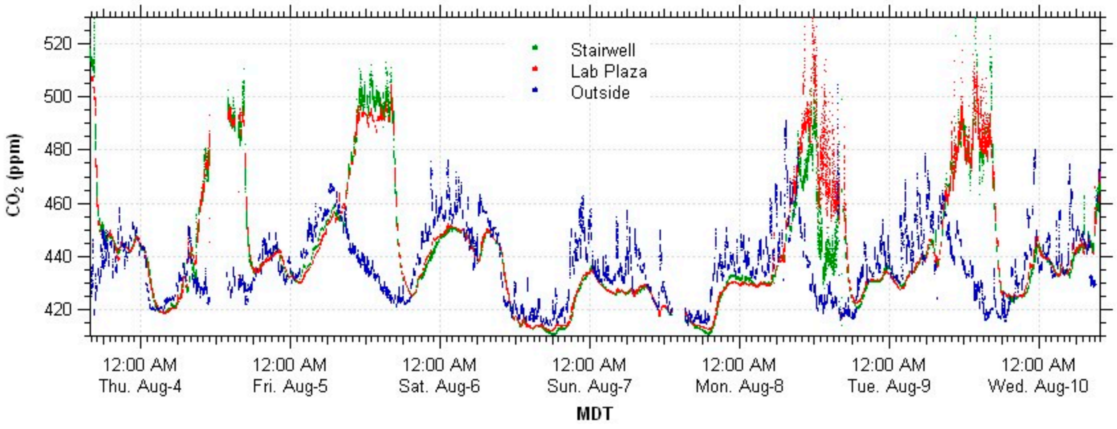

Figure S32. Summer 2005: University Building, Boulder (week 2, coincides with AMS data).

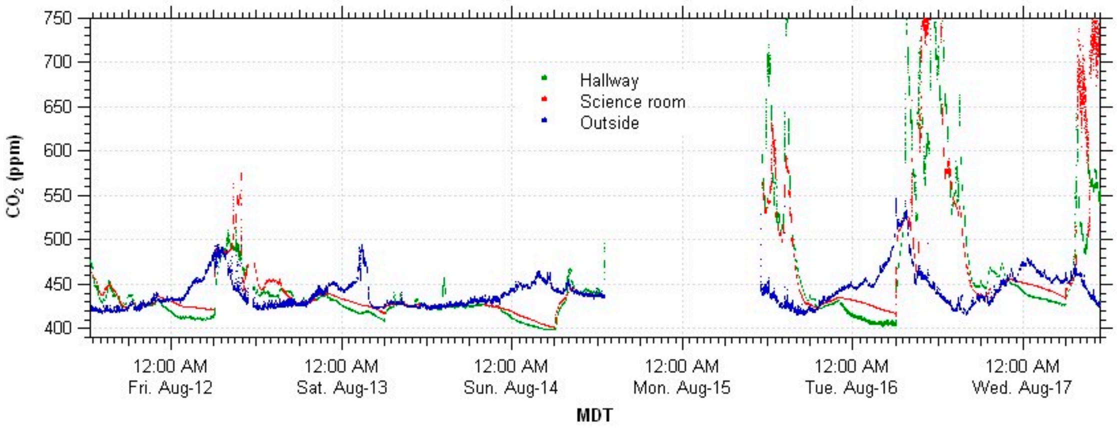

Figure S33. Summer 2005: Denver School (week 1, coincides with AMS).

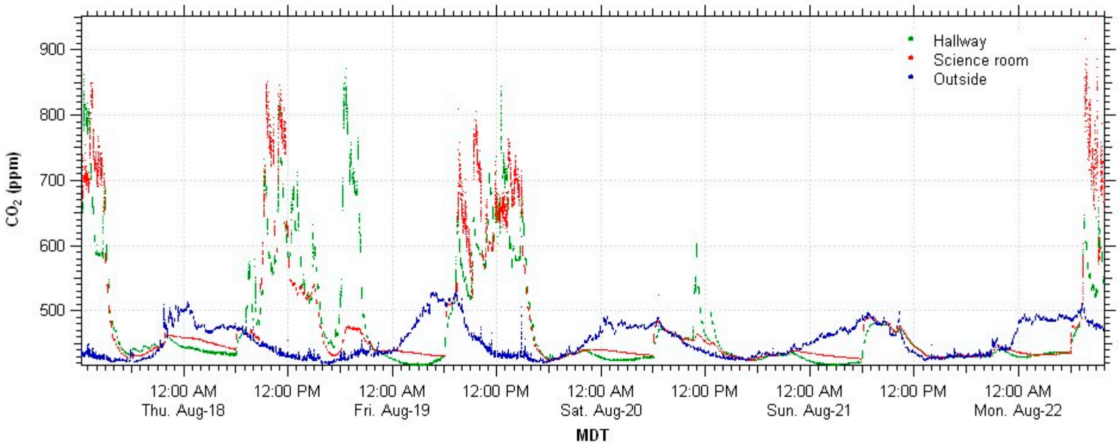

Figure S34. Summer 2005: Denver School (week 2, coincides with UHSAS).

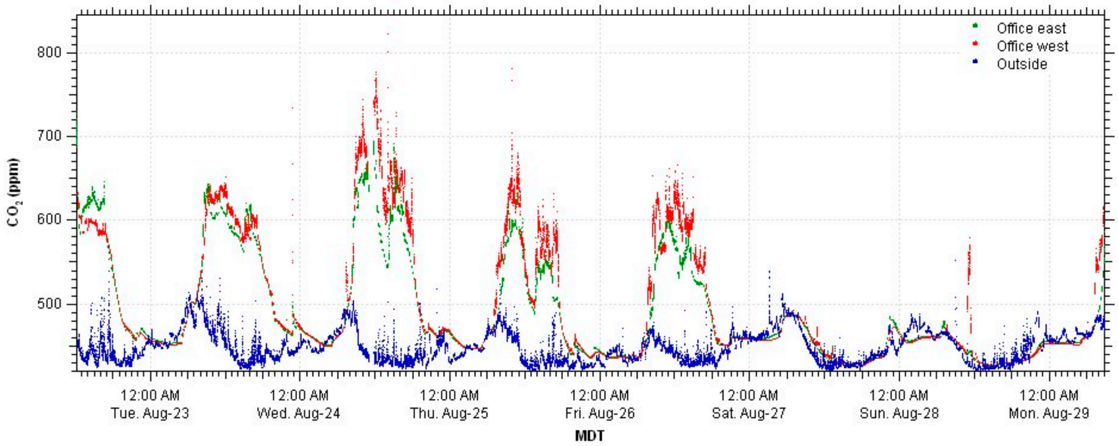

Figure S35. Summer 2005: Office building, Denver.

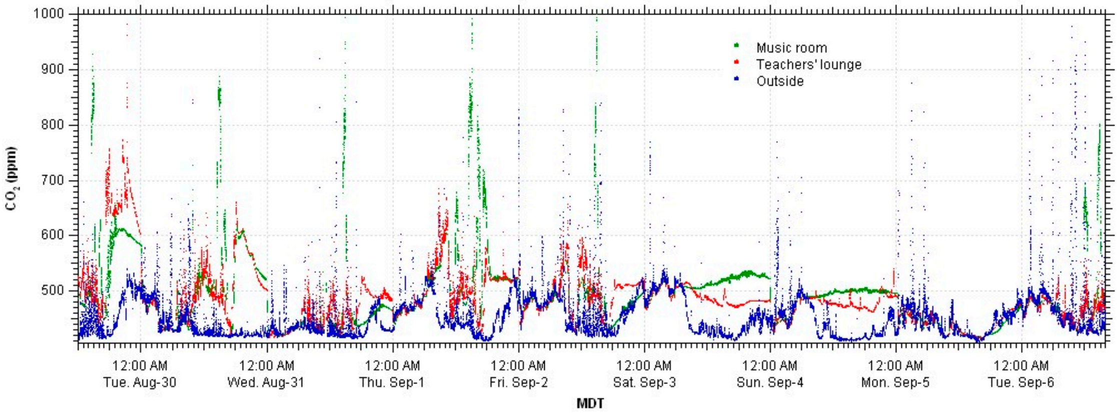

Figure S36. Summer 2005: Boulder School.

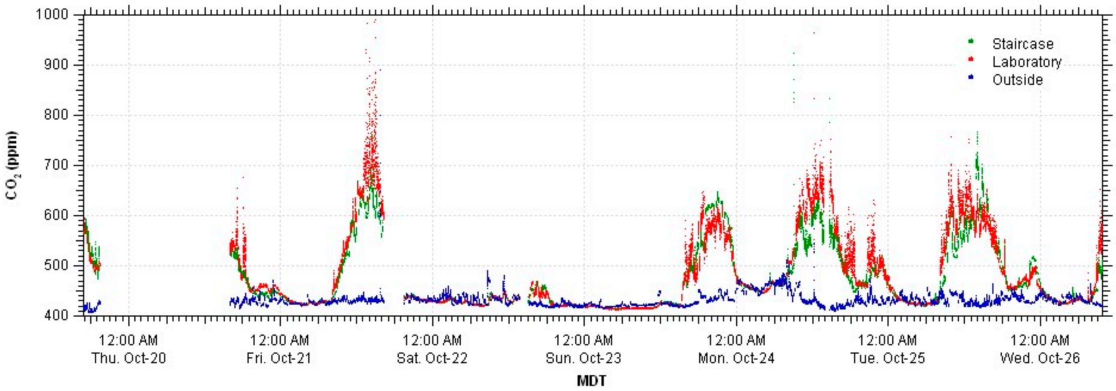

Figure S37. Fall 2005: University Building, Boulder.

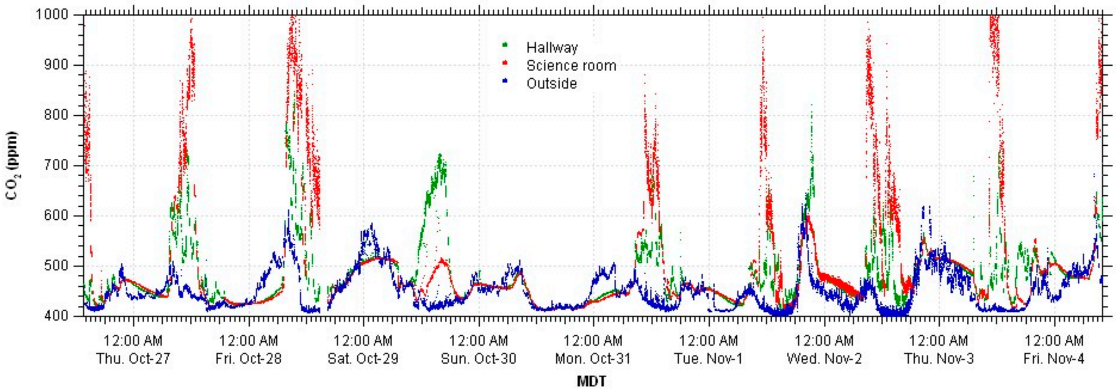

Figure S38. Fall 2005: Denver School.

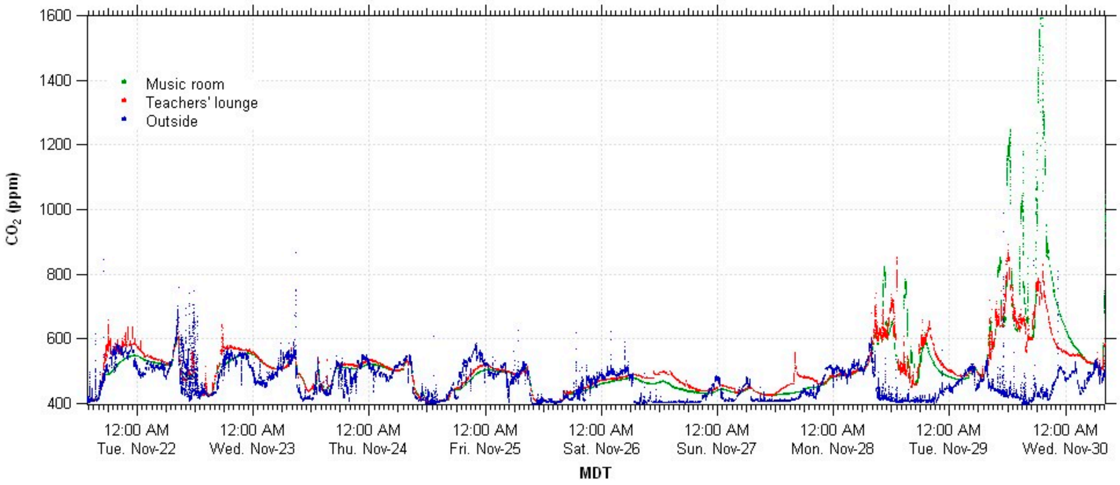

Figure S39. Fall 2005: Boulder School.

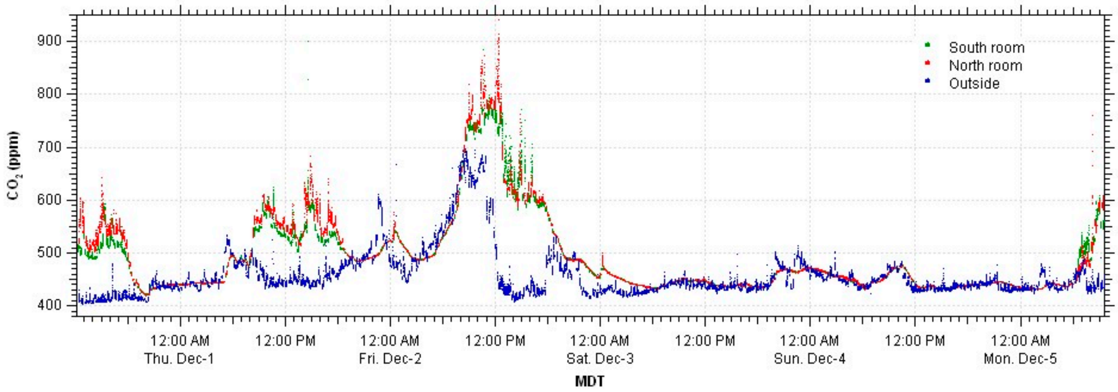

Figure S40. Fall 2005: Office building, Denver.

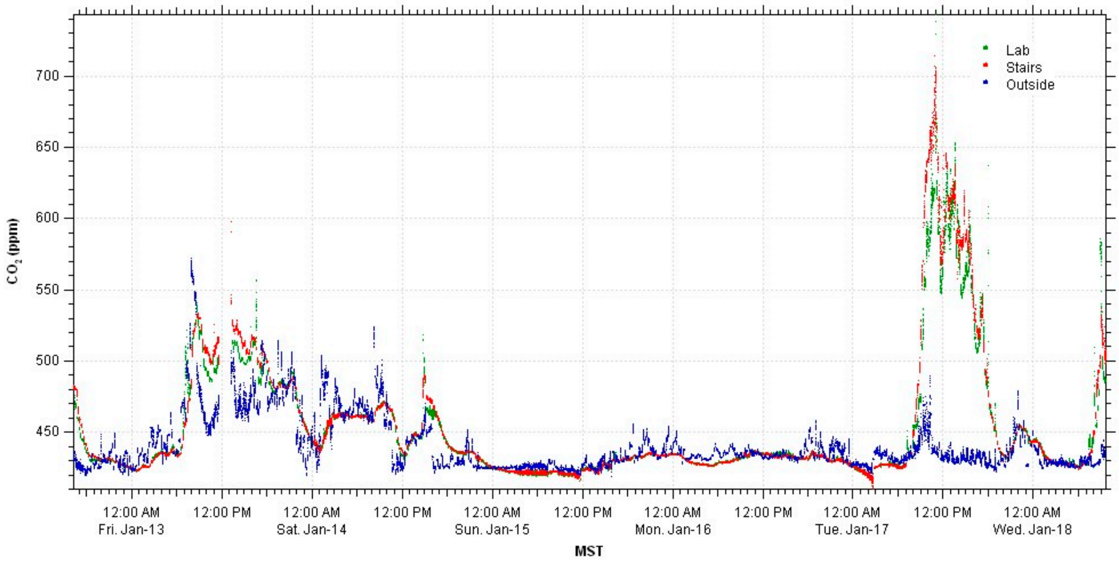

Figure S41. Winter 2006: University Building, Boulder.

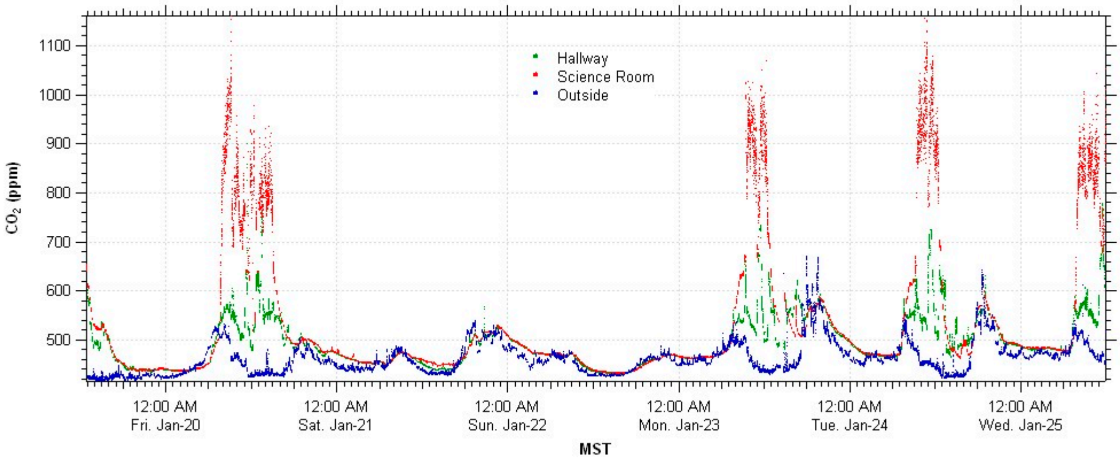

Figure S42. Winter 2006: Denver School.

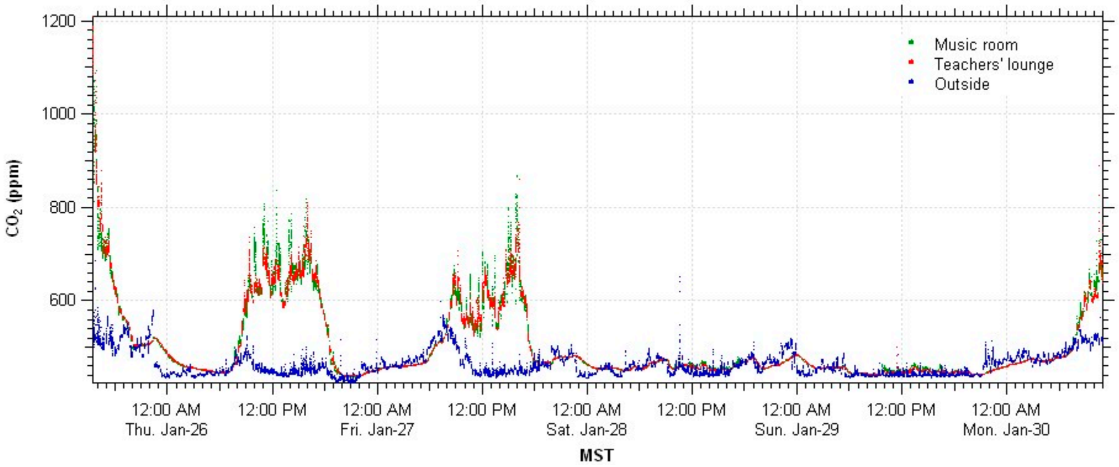

Figure S43. Winter 2006: Office building, Denver.

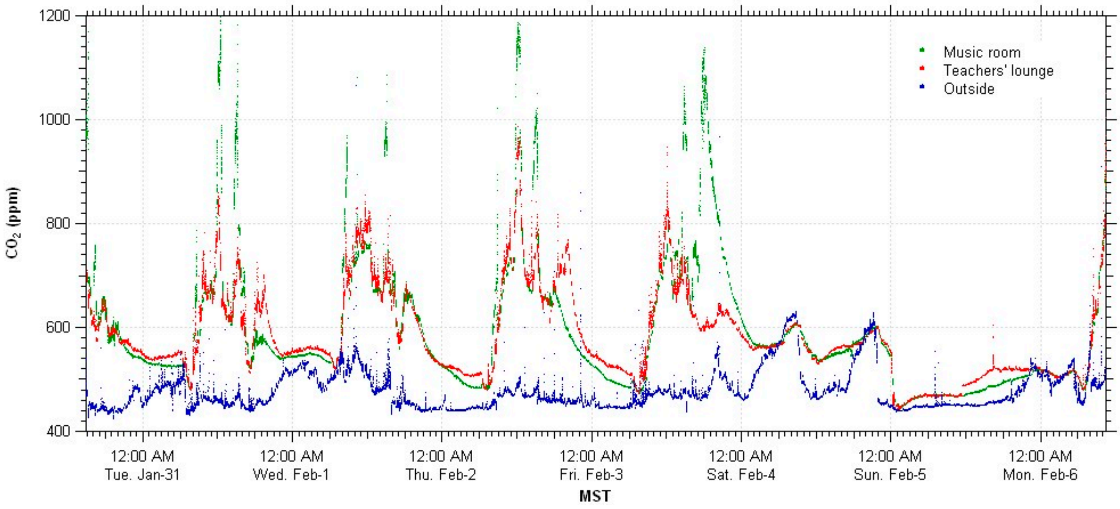

Figure S44. Winter 2006: Boulder School.

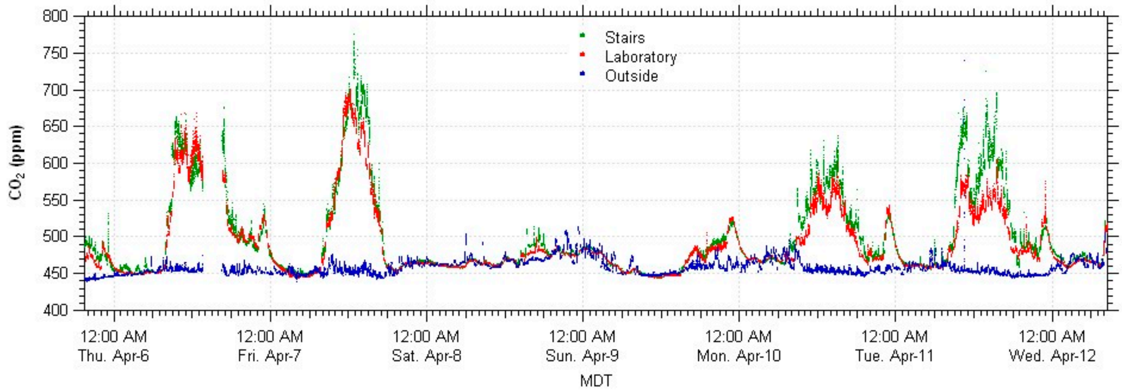

Figure S45. Spring 2006: University Building, Boulder.

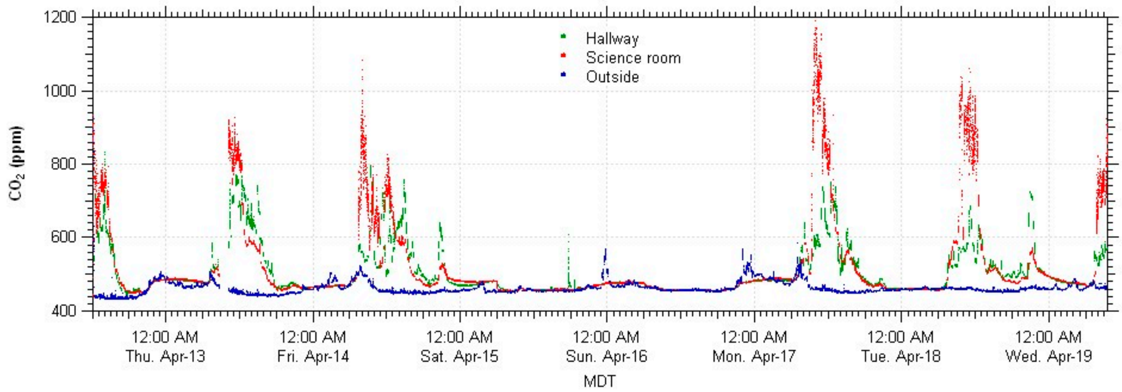

Figure S46. Spring 2006: Denver School.

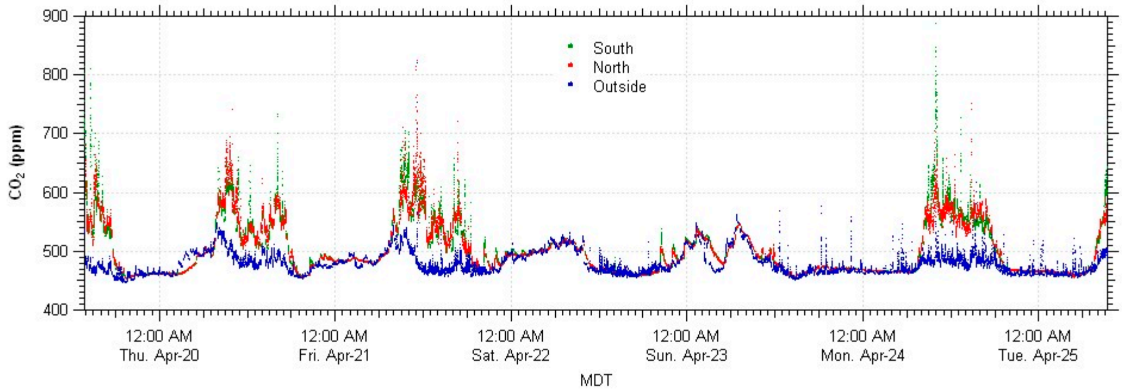

Figure S47. Spring 2006: Office building, Denver.

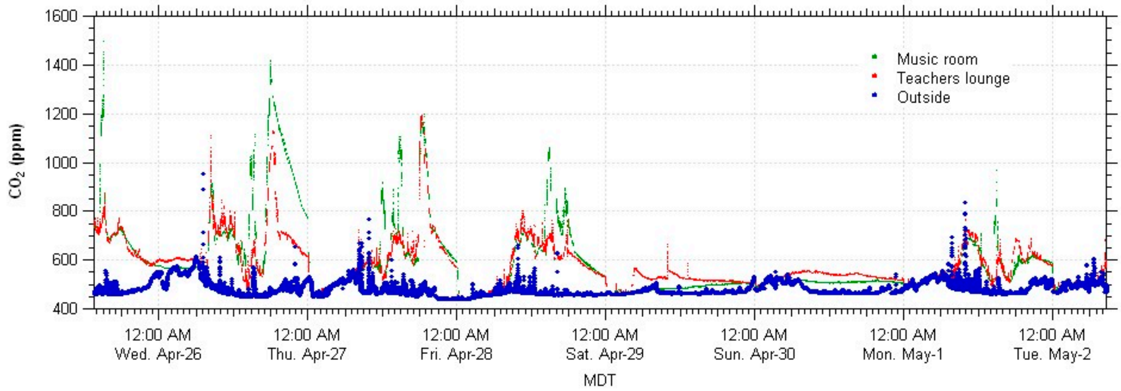

Figure S48. Spring 2006: Boulder School.

5. Section E: Tables of Average Lag Times and Standard Deviations

Table S9. Average (SD) lag times for each building.

Building Type	Day Type	Time of Day	Lag Times, min. Ultrafine	Fine
University		All	63 (63)	72 (114)
	Weekday	Day	9 (6)	15 (6)
		Night	33 (25)	30 (37)
	Weekend	Day	100 (57)	56 (43)
		Night	68 (55)	88 (67)
Denver School		All	24 (17)	15 (7)
	Weekday	Day	12 (0)	12 (6)
		Night	69 (130)	99 (184)
	Weekend	Day	21 (20)	9 (0)
		Night	93 (170)	138 (236)
Office		All	30 (7)	33 (29)
	Weekday	Day	15 (6)	18 (10)
		Night	36 (14)	21 (34)
	Weekend	Day	21 (6)	33 (6)
		Night	48 (26)	57 (17)
Boulder School		All	56 (42)	24 (42)
	Weekday	Day	8 (7)	24 (7)
		Night	44 (28)	32 (37)
	Weekend	Day	48 (48)	44 (48)
		Night	44 (28)	104 (32)

All = All data from that building; Day = 6a m–8p m; Night = 8 pm–6 am.

Table S10. Average (SD) lag times for each season.

Season	Day Type	Time of Day	Lag Times, min. Ultrafine	Fine
Summer		All	24 (12)	44 (35)
	Weekday	Day	16 (7)	16 (7)
		Night	124 (121)	152 (195)
	Weekend	Day	64 (69)	28 (18)
		Night	152 (171)	212 (233)
Fall		All	39 (18)	24 (17)
	Weekday	Day	9 (6)	12 (10)
		Night	24 (26)	21 (42)
	Weekend	Day	56 (37)	40 (39)
		Night	44 (18)	44 (18)
Winter		All	51 (32)	51 (32)
	Weekday	Day	9 (6)	6 (7)
		Night	39 (27)	27 (25)
	Weekend	Day	12 (17)	12 (10)
		Night	39 (28)	30 (29)
Spring		All	51 (70)	75 (118)
	Weekday	Day	12 (0)	12 (0)
		Night	15 (6)	27 (30)
	Weekend	Day	51 (48)	36 (49)
		Night	39 (62)	42 (69)

All = All data from that season; Day = 6 am–8 pm; Night = 8 pm–6 am.

Table S11. Average (SD) lag times for period of the week.

Day Type	Time of Day	Lag Times, min.	
		**Ultrafine**	**Fine**
Weekday	All	42 (37)	49 (60)
	Day	11 (5)	11 (7)
	Night	46 (62)	50 (91)
Weekend	Day	44 (43)	28 (30)
	Night	64 (86)	75 (119)
Weekday	Day	28 (47)	31 (68)
	Night	54 (69)	52 (90)
Weekend	Day	27 (34)	19 (23)
	Night	55 (75)	63 (106)

All = All data; Day = 6 am–8 pm; Night = 8 pm–6 am.

Table S12. Seasonal Average (SD) lag times for university building only, by HVAC usage.

Season	HVAC Usage	Lag Times, min.	
		**Ultrafine**	**Fine**
Summer	on	12	12
	off	48	60
Fall	on	24	12
	off	0	0
Winter	on	12	24
	off	48	36
Spring	on	12	0
	off	156	240
Total	on	15 (5)	12 (8)
	off	63 (57)	84 (93)

Standard deviations in parenthesis, where applicable; Total = all seasons combined.

6. Section F: Outdoor Hour-Averaged AMS Chemical Data Plots

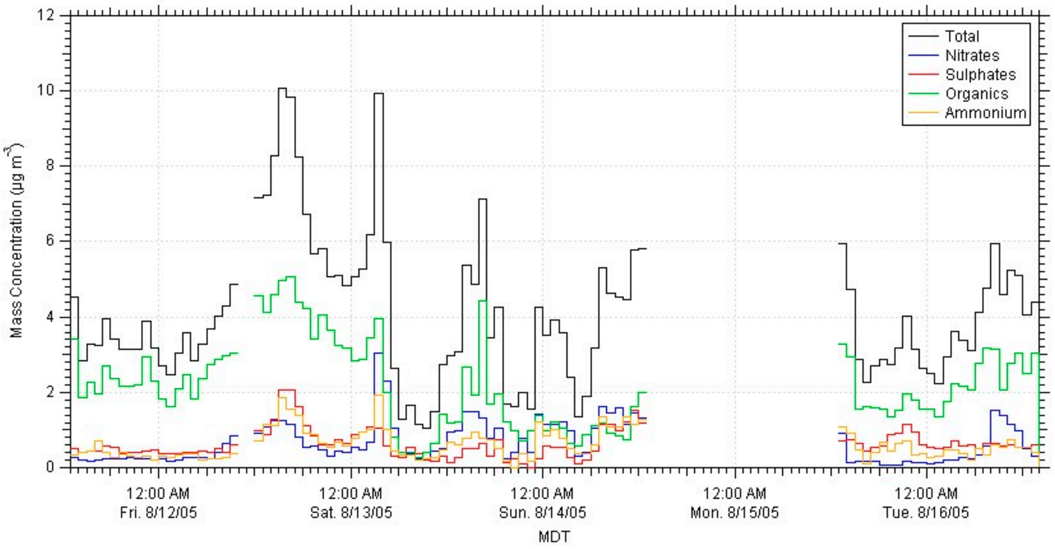

Figure S49. Summer 2005: Denver School.

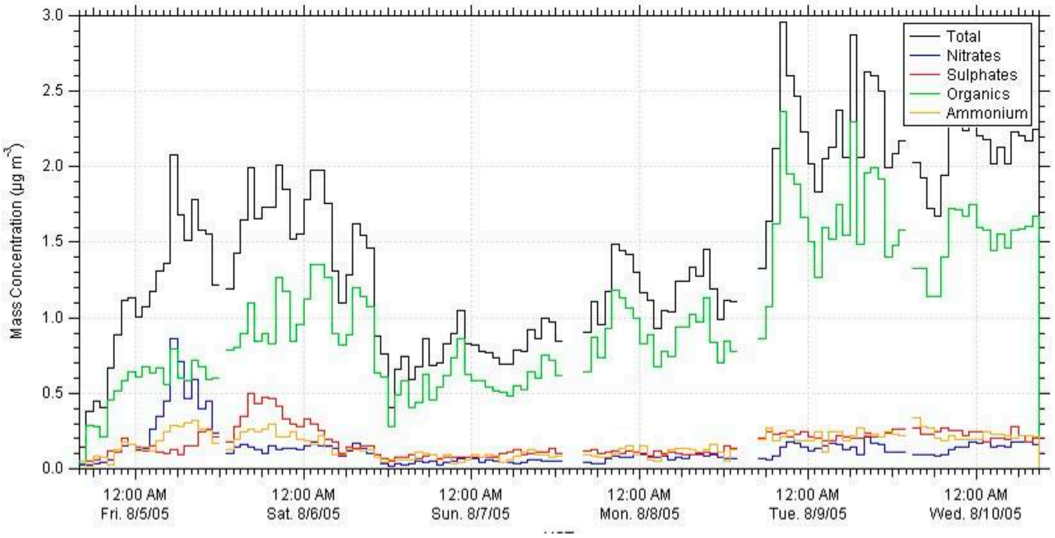

Figure S50. Summer 2005: University Building, Boulder.

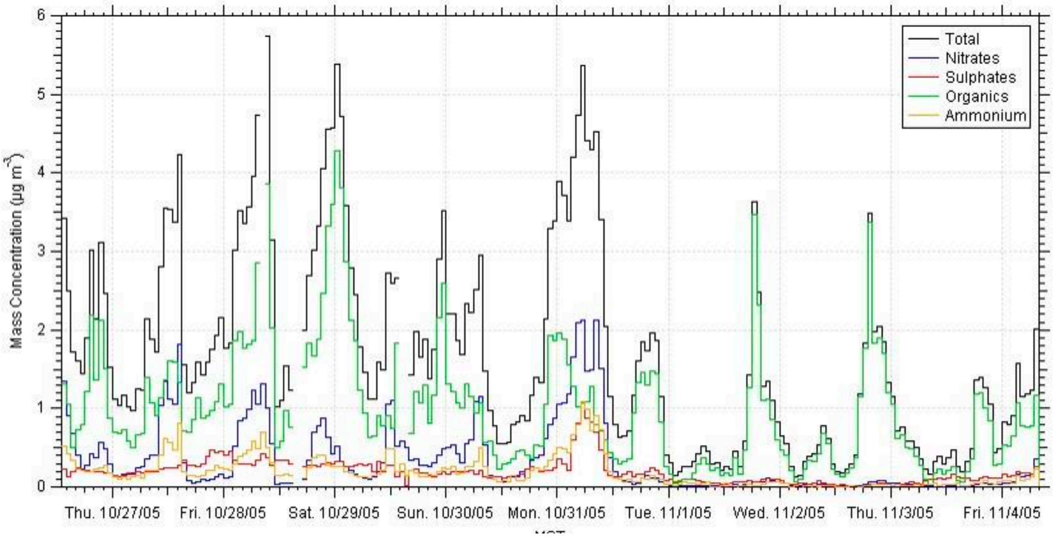

Figure S51. Fall 2005: Denver School.

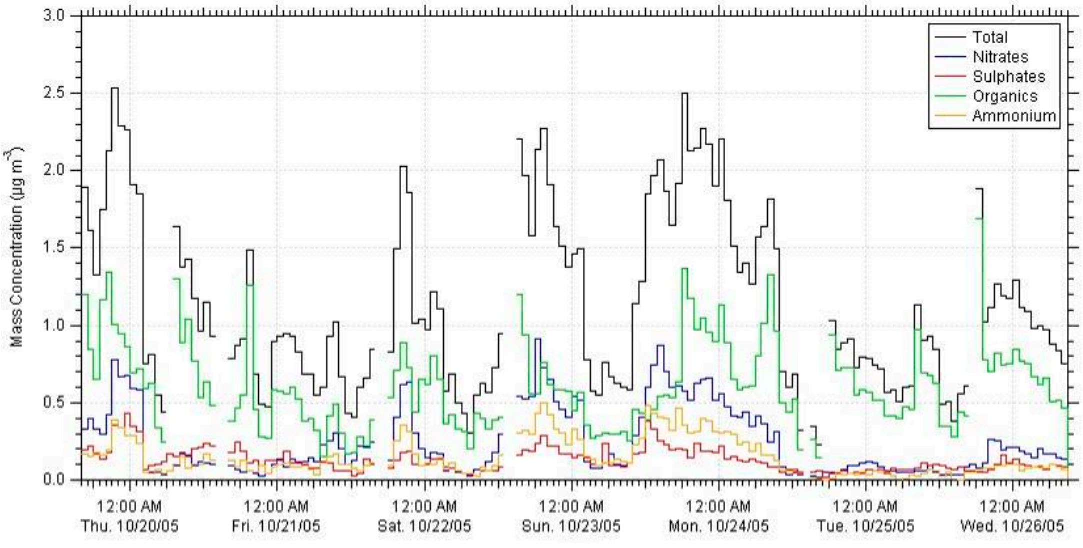

Figure S52. Fall 2005: University Building, Boulder.

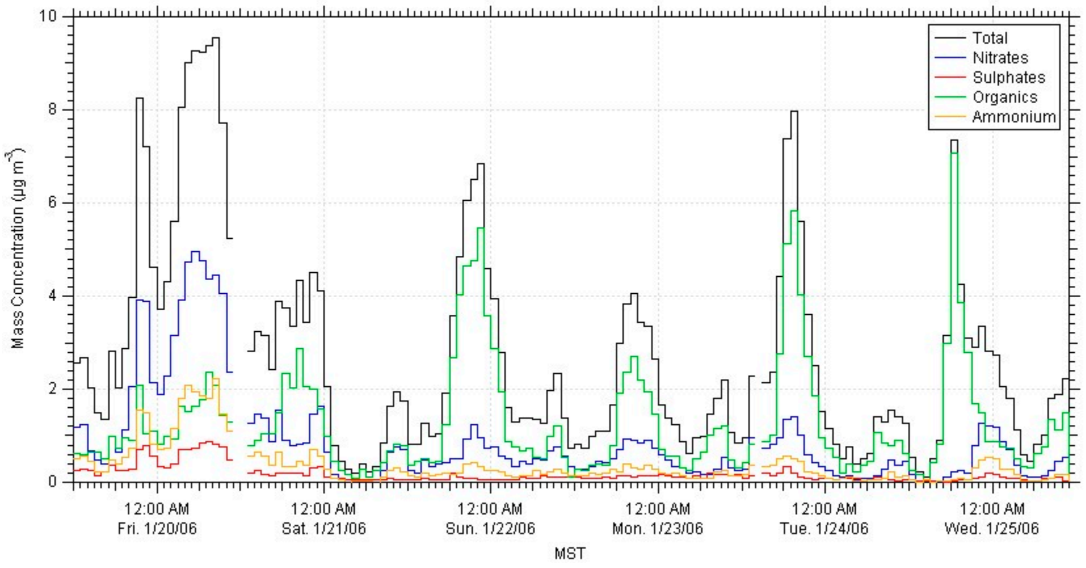

Figure S53. Winter 2006: Denver School.

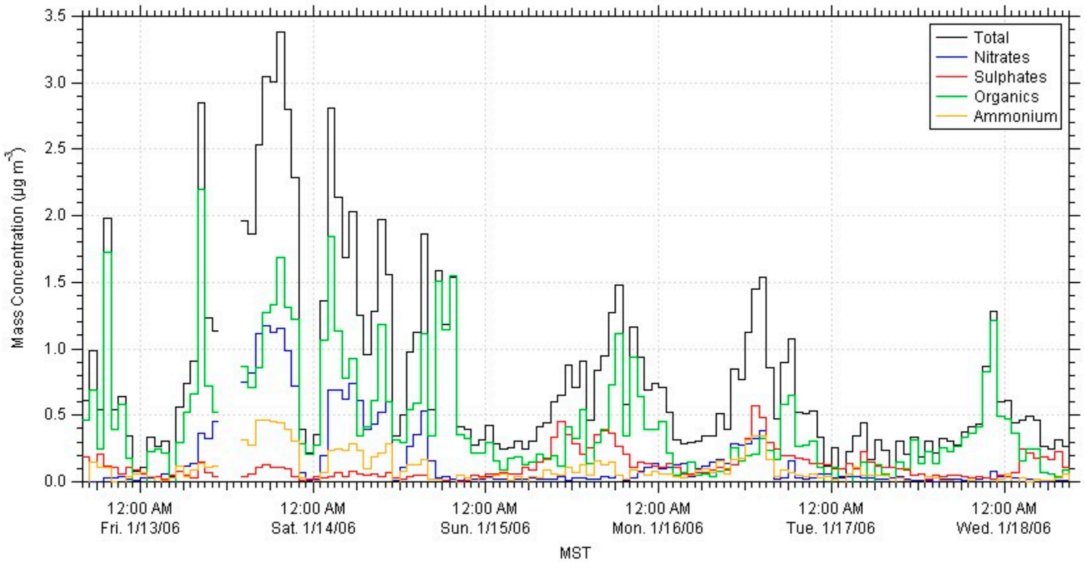

Figure S54. Winter 2006: University Building, Boulder.

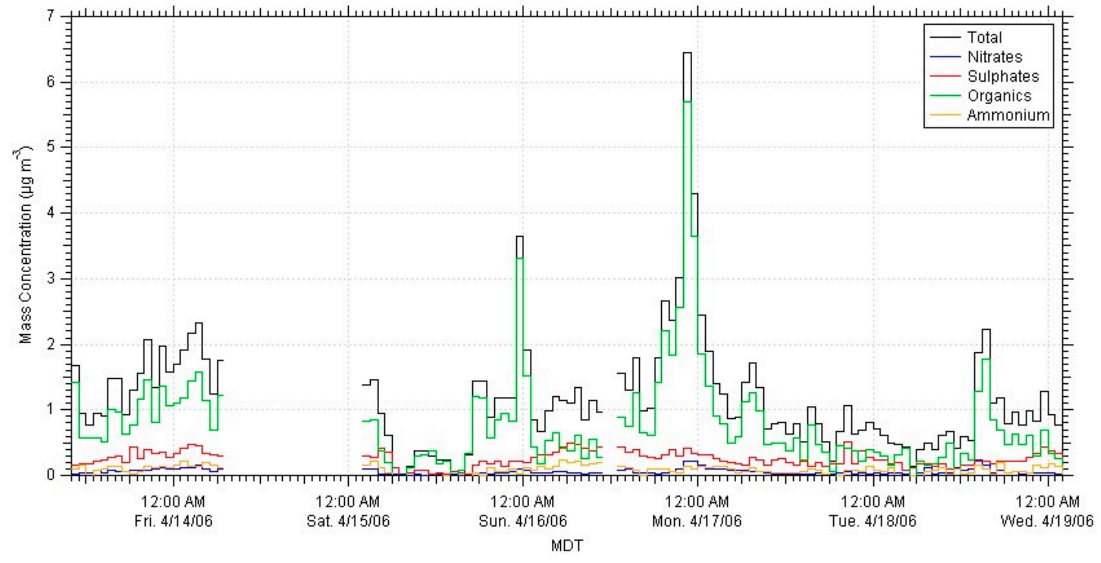

Figure S55. Spring 2006: Denver School.

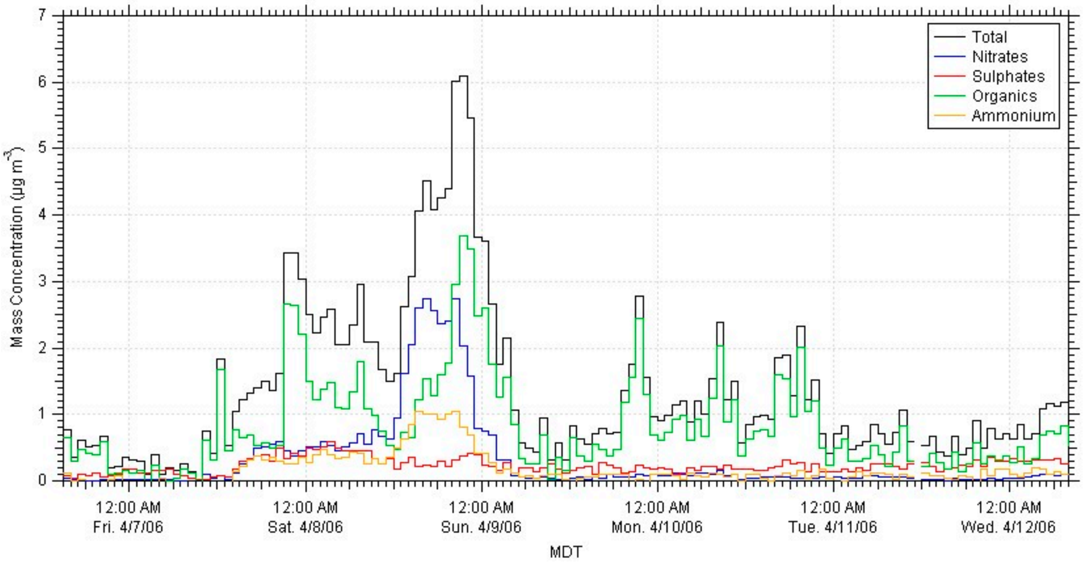

Figure S56. Spring 2006: University Building, Boulder.

© 2017 by the authors; licensee MDPI, Basel, Switzerland. This article is an open access article distributed under the terms and conditions of the Creative Commons Attribution (CC-BY) license (http://creativecommons.org/licenses/by/4.0/).
